# Deepening the knowledge of universal stress proteins in *Haloferax mediterranei*

**DOI:** 10.1007/s00253-023-12899-1

**Published:** 2024-01-16

**Authors:** Laura Matarredona, Basilio Zafrilla, Esther Rubio-Portillo, María-José Bonete, Julia Esclapez

**Affiliations:** 1 Department of Biochemistry and Molecular Biology and Soil Science and Agricultural Chemistry, Faculty of Science, University of Alicante, Ap 99, 03080 Alicante, Spain; 2 Department of Physiology, Genetics and Microbiology, Faculty of Science, University of Alicante, Ap 99, 03080 Alicante, Spain

**Keywords:** USP, RNA-Seq, Stress, Archaea

## Abstract

**Abstract:**

Haloarchaea, like many other microorganisms, have developed defense mechanisms such as universal stress proteins (USPs) to cope with environmental stresses affecting microbial growth. Despite the wide distribution of these proteins in Archaea, their biochemical characteristics still need to be discovered, and there needs to be more knowledge about them focusing on halophilic Archaea. Therefore, elucidating the role of USPs would provide valuable information to improve future biotechnological applications. Accordingly, transcriptional expression of the 37 annotated USPs in the *Haloferax mediterranei* genome has been examined under different stress conditions. From a global perspective, finding a clear tendency between particular USPs and specific stress conditions was not possible. Contrary, data analysis indicates that there is a recruitment mechanism of proteins with a similar sequence able to modulate the *H. mediterranei* growth, accelerating or slowing it, depending on their number. In fact, only three of these USPs were expressed in all the tested conditions, pointing to the cell needing a set of USPs to cope with stress conditions. After analysis of the RNA-Seq data, three differentially expressed USPs were selected and homologously overexpressed. According to the growth data, the overexpression of USPs induces a gain of tolerance in response to stress, as a rule. Therefore, this is the only work that studies all the USPs in an archaeon. It represents a significant first base to continue advancing, not only in this important family of stress proteins but also in the field of biotechnology and, at an industrial level, to improve applications such as designing microorganisms resistant to stress situations.

**Key points:**

*• Expression of Haloferax mediterranei USPs has been analyzed in stress conditions.*

*• RNA-seq analysis reveals that most of the USPs in H. mediterranei are downregulated.*

*• Homologous overexpression of USPs results in more stress-tolerant strains.*

**Supplementary Information:**

The online version contains supplementary material available at 10.1007/s00253-023-12899-1.

## Introduction

Increasing environmental alterations due to climate change and anthropogenic activities are causing a substantial impact on microbial communities worldwide. Many microorganisms require optimum growth conditions and cannot handle thrilling environmental conditions. Extreme conditions of temperature, salinity, pH, heavy metals, and oxidative stress, among others, affect the natural conformation of cells by inducing stress protein expression that plays a vital role in maintaining cell homeostasis. In addition, these problematic conditions can alter the native conformation of proteins, thus losing their function, which is the most relevant effect caused by these stressors. To cope with these conditions and protect cells, halophilic Archaea has developed several strategies, such as heat shock and stress proteins, molecular chaperones, thermoprotectants, multicellular structures, and other stress proteins (Matarredona et al. 2020).

An important superfamily of proteins, which are overexpressed against abrupt environmental changes, is the Universal Stress Protein (USP). This family is widely distributed in bacteria, plants, metazoans, yeast, fungi, invertebrates, and protist, that is to say, in the three Domains of life, being an essential part of their defense mechanisms against a multitude of environmental stresses such as heavy metal toxicity, nutrient starvation, heat/cold shock and ethanol (Kvint et al. 2003; Liu et al. 2007; Wang et al. 2017; Vollmer and Bark 2018; Ye et al. 2020). At a structural level, USPs of halophilic archaea can present different functional domains, as a single USP domain, a tandem USP domain, or with one or two USP domains fused to other catalytic motifs, such as amino acid permease or a Na^+^/H^+^ exchanger (Matarredona et al. 2020). The USP domain is known to provide the organism with the ability to respond to various environmental stresses.

This protein family can be divided into two groups, due to the presence of the ATP-binding domain, as in *Methanocaldococcus jannaschii,* which may be a molecular mechanism for regulating its function, or non-ATP-binding, represented by UspA from *Escherichia coli* and *Haemophilus influenzae* (Zarembinski et al. 1998; Sousa and McKay 2001; Kvint et al. 2003; Vollmer and Bark 2018).

In the past decades, the knowledge of this family of proteins has been widely studied to explore physiological functions in bacteria and plants; however, more is needed in archaea. In bacteria, these stress proteins have been found to play a role in various situations, such as DNA damaging agents, respiratory uncouplers, resistance to oxidative stress, adhesion, biofilm formation, motility, bacterial infection, transport, scaffolding and signaling (Kvint et al. 2003; Nachin et al. 2005; O’Connor and McClean 2017; Vollmer and Bark 2018). These are just some of the functions they can perform, as many more have been found. It is similar in plants, as there is a large amount of work studying the benefits of these proteins in the face of abiotic stresses, such as oxidative stress, drought, osmotic stress, and the absence of oxygen (Gonzali et al. 2015; Gutiérrez-Beltrán et al. 2017; Vollmer and Bark 2018).

As described above, there needs to be more information on the function of these stress proteins in the archaeal Domain; in fact, only two papers have been found in archaeal species. One large study reported that *Sa*UspA plays a role in nutrient starvation and adaptation to high salinities in response to hyper- and hypo-shock treatments in *Sulfolobus acidocaldarius* and *Methanohalophilus portucalensi* (Shih and Lai 2010; Ye et al. 2020). To extend the knowledge of this family of proteins, it was decided to study the halophilic archaea *Haloferax mediterranei* ATCC 33500^ T^, which has revealed 37 genes encoding USP domain-containing proteins. USP expression depends on the type of stress and growth phase. Therefore, transcriptional expression analysis of all identified USPs was performed under standard and stress conditions by reverse transcription polymerase chain reaction (RT-PCR). Four of these conditions were chosen to analyze the expression levels of the 37 USPs by transcriptome sequencing (RNA-Seq). After that, three USPs were homologously overexpressed and purified under native conditions; the overexpression strains were also characterized using different stress conditions.

Overall, this study provides fundamental information on the behavior of universal stress proteins in *H. mediterranei* under different stress conditions and provides information to classify these proteins into other groups. It offers the first comprehensive analysis of all USPs annotated in a halophilic microorganism. This evidence will improve our understanding of the stress response of haloarchaea and provide an excellent starting point for elucidating the function of these proteins in halophiles.

## Materials and methods

### Bioinformatic analysis

Following the determination of amino acid sequence, protein structure, and phylogenetic relationships, a bioinformatic analysis was performed to study the USPs in *H. mediterranei* in depth. A phylogenetic tree was constructed using 37 USP sequences obtained from the protein database of NCBI (National Center for Biotechnology Information) (https://www.ncbi.nlm.nih.gov/protein/, accessed on 13 April 2023). The multiple alignments were performed using the software Clustal Omega (ClustalO) (https://www.ebi.ac.uk/Tools/msa/clustalo/, accessed on 21 April 2023) based on the HH algorithm described by Söding (Sievers et al. 2011; Letunic and Bork 2019). Then, the phylogenetic tree was built using the neighbor-joining method from Clustal Omega. The display, manipulation, and annotation of the phylogenetic tree were done using the online tool known as Interactive Tree Of Life (iTol) v4 (https://itol.embl.de/, accessed on 13 October 2021) (Letunic and Bork 2019).

The UniProt database (https://www.uniprot.org/, accessed on 21 April 2023) was used to analyze the domain structures. ClustalO-generated multiple sequence alignment was used to determine the presence of ATP-binding amino acid residues, using USP (MJ_0577) from *M. jannaschi* as a template.

### Strains and standard growth conditions

*E. coli* strains DH5α and JM110 for cloning and preparing unmethylated DNA, respectively. Cells were grown overnight in Luria–Bertani medium with ampicillin (100 µg/mL) at 37 °C.

*H. mediterranei* R4 (ATCC 33500^ T^) was grown in a complex medium (Hm-CM) containing 20% (w/v) mixture of inorganic salts (seawater, SW) and 0.5% (w/v) yeast extract (pH 7.3); and defined medium (Hm-DM) contained a concentration of 20% (w/v) seawater and 20 mM NH_4_Cl (pH 7.3). After autoclaving and cooling, it was supplemented with 0.03 mM FeCl_3_, 1 mM KH_2_PO_4_, 7.5 mM CaCl_2,_ and 50 mM MOPS (3-(N-morpholino)propane sulfonic acid). This culture medium is used as the control in this work.

The overexpression strains (USP5-HM26, USP21-HM26, and USP28-HM26) were performed using *H. mediterranei* HM26 (R4 Δ*pyrE*2) grown in minimal media (Hm-MM) (Matarredona et al. 2021a).

### Induced stress conditions

*H. mediterranei* was subjected to different stresses (Table 1) to analyze the transcriptional expression of USPs under salinity, pH, temperature, hydrogen peroxide, carbon starvation, nitrogen starvation, and the presence of metals that are toxic for growth (Li^+^, Co^2+^, As^5+^, and Ni^2+^). Hm-DM was used as basal media in almost all tested conditions, varying in each one only a parameter. The control medium with ammonium behaves like the complex medium. All culture media were inoculated at optical density at 600 nm wavelength (OD_600_) 0.02 with pre-adapted cells. Cells were collected in each culture media's initial exponential, mid-exponential, and stationary phases. Cell growth was quantified by measuring the OD_600_ at regular intervals until the stationary phase. Three independent biological replicates of each condition were performed.

**Table 1:** Culture media and stress conditions used to analyze the USPs expression in *H. mediterranei*

Study variable	Culture media composition
Control condition	Hm-DM containing 20% SW and grown at 42 ºC and pH 7.2
Temperature	Hm-DM cultures were grown at 32 ºC and 52 ºC
Salinity	Hm-DM cultures containing 12.5% and 32.5% SW
pH	The pH of Hm-DM cultures was adjusted to 6.26 and 8.25
Oxidative stress *	Hm-DM cultures were grown to OD_600_ of 0.8 (mid-exponential phase) before adding 8 mM H_2_O_2_
Metal stress *	Hm-DM cultures containing 0.4 mM nickel (Ni^2+^); 0.2 mM cobalt (Co^2+^); 2 mM arsenic (As^5+^); and 0.5 M lithium (Li^+^)
Nitrogen starvation	Hm-NS 24, 48 and 72 h (Matarredona et al. 2021a)
Carbon starvation	Hm-CS 24, 48 and 72 h (Matarredona et al. 2021a)

* The hydrogen peroxide and heavy metals cultures were performed as Matarredona et al. (2021b)

### Reverse transcription-polymerase chain reaction (RT-PCR) analysis

The expression of all annotated USPs was analyzed by RT-PCR using RNA from cells cultivated under different conditions and growth phases. Total RNA was isolated from *H. mediterranei* R4 using an RNeasy Mini kit (Qiagen, Stockach, Germany) following the manufacturer's instructions. Afterward, RNA samples were treated with Turbo DNase (Invitrogen, Waltham, Massachusetts, USA) and analyzed as described in Esclapez et al. (2015). All samples showed an RNA integrity number (RIN) higher than 8. Total RNA (0.8 μg) was reverse transcribed into cDNA following the products specifications (Thermo Fisher Scientific, Waltham, Massachusetts, USA). The sequences of the specific oligonucleotide primers were designed to target a 120 bp region of the gene (Table S1). RT-PCR amplicons were analyzed using 1.5% (w/v) agarose gel electrophoresis and GeneRuler™ Low Range DNA Ladder (Thermo Scientific, Waltham, Massachusetts, USA). The output data were structured into binary tables indicating the absence (0) or presence (1) of a USP transcript under specific condition and at relevant growth time points. To emphasize differences between the expression patterns of a stressful condition and the control condition, their corresponding values (0 or 1) were subtracted. In the resulting output tables, zero values indicated no changes in the USP expression, while 1 and -1 meant the presence or absence of a specific UPS in a particular condition compared to the control, respectively.

### RNA-Seq

Culture conditions for RNA-Seq were: Hm-DM in the presence of 20 mM ammonium (control condition), Hm-DM with 8 mM H_2_O_2_, Hm-CS at 48 h, and 0.5 M lithium (LiCl). Three independent replicates of *H. mediterranei* R4 cells from each condition were grown, harvested, and RNA was isolated as detailed above. Following this, the transcriptome of 12 samples was analyzed by RNA-Seq (Macrogen Inc. Seoul, Korea). The RNA-Seq library preparation and sequencing were performed using the TruSeq Stranded Total RNA with NeBNext rRNA Depletion Kit (New England BioLabs, Ipswich, Massachusetts, USA) to construct the cDNA libraries and subsequently sequenced using an Illumina NovaSeq 6000 system (Illumina, Cambridge, United Kingdom) according to manufacturer's guidelines. The libraries were checked and quantified using an Agilent Technologies 2100 Bioanalyzer by qPCR following qPRC Quantification Protocol Guide (Agilent, Santa Clara, USA).

### Analysis of gene expression and detection of differentially expressed genes

Raw reads were cleaned by trimming the adaptor sequences and low-quality ends (quality score > 30) using PRINSEQ (Schmieder and Edwards 2011), and ribosomal reads were identified using RNAscan (Le et al. 2004). Forward clean reads were mapped against genomes using TopHat 2.1.1 (Kim et al. 2013) and counted using HTSeq 0.6.1 (Anders et al. 2015). The expression profiles were normalized using the number of reads and the ORF length, and these values were provided as reads per kilobase pair of transcripts per million mapped reads (RPKM). Differentially expressed genes, defined as genes whose expression levels differed more than twofold between the treatment and the control conditions, and for which the p-value was < 0.001, were identified using DESeq2 (Love et al. 2014), a variance analysis package that was developed to infer statistically significant differences in gene expression data from high-throughput sequencing. Data from replicates from the same condition were highly reproducible, showing Pearson coefficient values between 0.877 and 0.997, except for replicate 2 from the H_2_O_2_ 8 mM treatment, which was eliminated from the analysis. Raw data are available on NCBI, Bioproject PRJNA988275.

### Homologous overexpression of USPs in *H. mediterranei* HM26

The *usp5*, *usp21,* and *usp28* genes were amplified from *H. mediterranei* R4 genomic DNA using the corresponding primers, which include the restriction sites: *usp5 (EcoR*I/ *BspH*I), *usp21* (*EcoR*I/ *pci*I) and *usp28* (*EcoR*I/ *Nco*I) (Thermo Fisher Scientific, Waltham, Massachusetts, USA) (Table S2). The three-plasmid constructions (pTA1992.*usp5*, pTA1992.*usp21,* and pTA1992.*usp28*) were generated using T4 DNA Ligase (Thermo Scientific, Waltham, Massachusetts, USA) retaining the vector's N-terminal His_6_ tag and following the same procedure as in Matarredona et al. (2021a). Following this, *H. mediterranei* HM26 cells were independently transformed with the three constructions, using polyethylene glycol 600 and grown on Hm-MM agar plates (Cline et al. 1989). Different colonies were grown into liquid media until the stationary phase; cells were collected by centrifugation at 13,000 rpm for 30 min and resuspended in binding buffer (20 mM Tris–HCl, 1.5 M NaCl, 50 mM imidazole, pH 7.4). Afterward, cells were lysed by sonication and centrifuged, and the supernatant was collected to perform the first chromatographic step.

### Protein purification and determination of molecular mass

Two chromatographic steps were performed on an ÄKTA chromatography system (GE Healthcare Life Sciences, Cornella de Llobregat, Spain) to obtain the three highly purified USP proteins. The first step was nickel affinity chromatography using a prepacked HisTrap HP 5 mL column (Cytiva, Cornella de Llobregat, Spain), following the manufacturer's indications. The elution buffer contained 20 mM Tris–HCl, 1.5 M NaCl, and 500 mM imidazole, pH 7.4. The second step was gel filtration chromatography using HiPrep 16/60 Sephacryl S-200 HR (Cytiva, Cornella de Llobregat, Spain), using the above binding buffer. All fractions were analyzed on 14% SDS-PAGE, using PageRuler Plus Prestained Protein Ladder (Thermo Fisher Scientific, Waltham, MA, USA) as molecular weight markers. Proteins were detected using Coomassie Brilliant Blue staining.

### Analysis of USPs overexpression effect on the *H. mediterranei *HM26 growth

Phenotypic characterization of the three overexpression strains (USP5-HM26, USP21-HM26, and USP28-HM26) and *H. mediterranei* HM26 was performed in the same culture media as the RT-PCR assay (Table 1) by measuring the optical density at 600 nm wavelength at regular intervals until the stationary phase was reached. The three phases of cell growth were compared over time. To ensure reproducibility, three independent biological replicates were performed for each condition. All cultures were inoculated at an initial optical density of 0.02 with cells previously grown in Hm-MM. The doubling time (d.t.) was calculated as Matarredona et al. (2021b).

Statistical analysis was performed using GraphPad Prism (Version 8), and values in the growth curves are expressed as the mean of the three biological replicates and the standard deviation.

## Results

### Sequence analysis and phylogenetic study of USPs in *H. mediterranei* R4

**Fig. 1:**
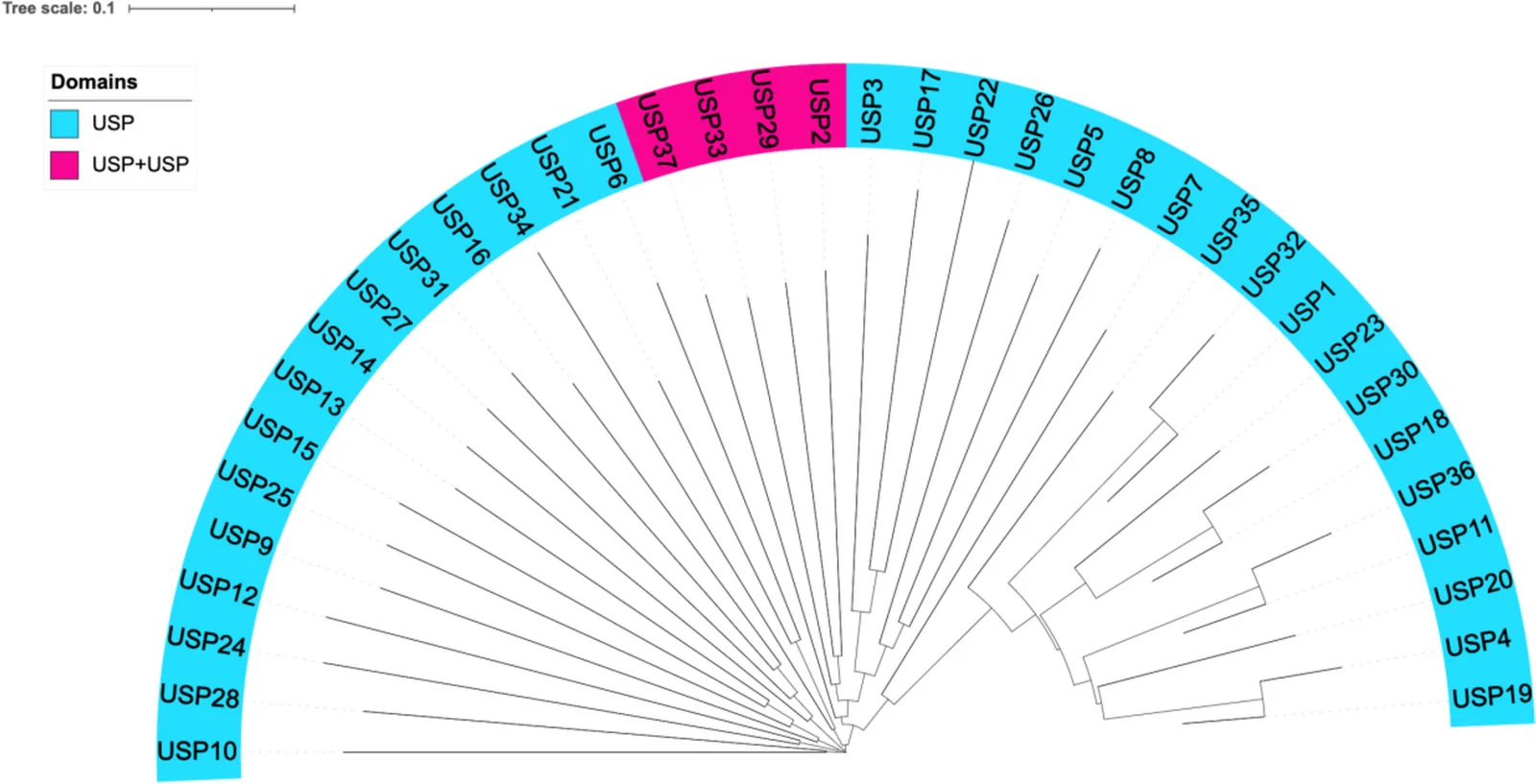
Phylogenetic tree of the 37 amino acid sequences of USPs proteins from *H. mediterranei,* including a description of their domain structures. The phylogenetic tree was built with Clustal Omega and iTol v4 software. In blue, USPs contain a single USP domain; in pink, USPs have two USP domains

Halophilic microorganisms encode many USP proteins in their genomes; consequently, it appears that these proteins play crucial roles in maintaining cell homeostasis under extreme external factors. In particular, *Haloferax* is one of the genera with the highest number of USPs identified, with a total of 1301, just behind the *Halorubrum* genus in which 1939 has been identified (Matarredona et al. 2020). The genome of *H. mediterranei* ATCC 33500^ T^ contains 37 USPs homologs proteins whose phylogeny and domain identification have been studied (Fig. 1). A phylogenetic tree was constructed to explore the phylogenetic relationships among the USP family members of *H. mediterranei* by analyzing 37 available USP protein-coding sequences. The left side of the phylogenetic tree shows a clear and early divergence of most branches, which is in line with what is expected due to the low sequence conservation of these proteins. The right side is not as divergent due to the similarity of amino acid residues. To understand in depth how 37 USP family members have diverged over the years, it would be helpful to know their function in the cell. The analysis of the amino acid sequences revealed that all *H. mediterranei* USPs contain at least one USP domain. This is the typical domain structure found in USP proteins and contains around 140–160 amino acids. Most USPs proteins in *H. mediterranei* contain a single 14–15 kDa domain constituting the entire protein. Only 4 of the 37 USPs proteins contain a tandem domain. Although some archaeal USPs are known to have the USP domain fused to another functional motif, such as amino acid permease or a Na^+^/H^+^ exchanger, these motifs are not conserved in all haloarchaea, as in the case of *H*. *mediterranei.* This family's average number of amino acid residues is around 150–180. However, the USPs that contain two USP domains differ in expected length with longer chains around 290 residues (USP2, USP29, USP33, and USP37 with 293, 290, 289, and 292, respectively). More detailed information about protein and domain length is shown in Table S3, revealing that the USP domain represents almost the entire sequence. These characteristics differ, for example, in other species of the same genus, such as *Haloferax gibbonsii*. This halophilic archaeon has not only a higher number of USPs annotated in its genome, but all of them contain one or two USP domains and a length of about 160 amino acids. However, *Halorubrum terrestre* has 46 annotated USPs containing the USP domain and around 150 amino acids. Therefore, it appears that *H. mediterranei* has characteristics that differ from other microorganisms.

**Fig. 2:**
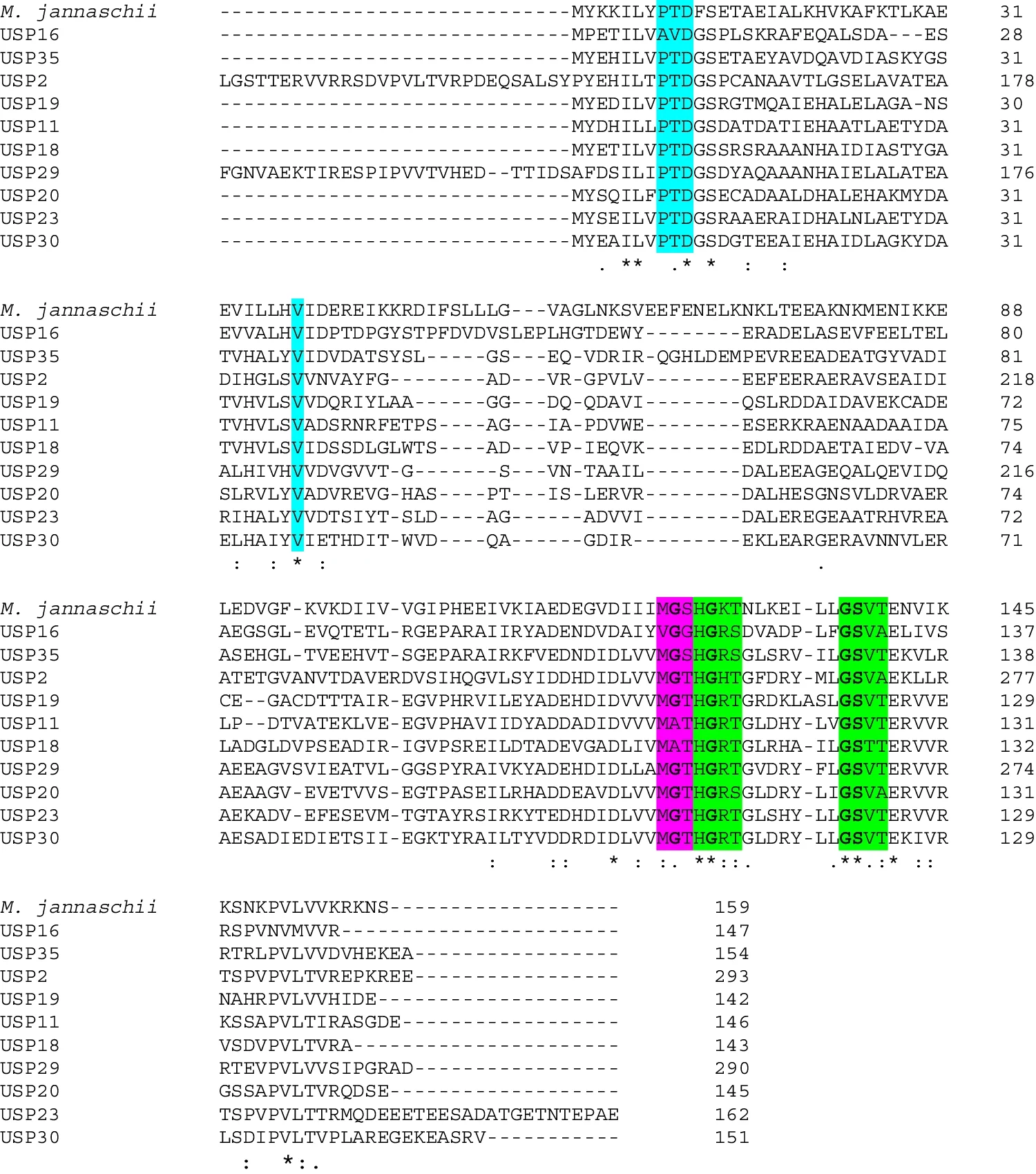
Multiple sequence alignment of USP proteins from *H. mediterranei* and MJ0577 from *M. jannaschii*. Conserved residues involved in ATP binding are shown in colours (Blue: residues involved in adenine binding; Green: Phosphate; Pink: Ribose). In bold, the motif G2xG9xG(S/T) contains the ATP motif. They were aligned using as reference an USP in *M. jannaschii* (Zarembinski et al. 1998)

The 37 *H. mediterranei* USPs have been classified into two subclasses according to the presence or absence of the ATP-binding motif in their amino acid sequences at the C-terminal region. To establish this classification, the 37 sequences of USPs were used to predict ligand binding sites based on alignments using a known ATP-binding universal stress protein MJ0577 from *M. jannaschii* as reference. The structure of this protein shows the motif G2xG9xG(S/T), which includes the ribosyl and phosphoryl groups of ATP; this motif was also present in USPs from *Schistosoma mansoni* (Isokpehi et al. 2011). The sequence alignment result is shown in Fig. 2, where amino acid residues contacting ATP are underlined in colors according to the annotation in Zarembinski et al. (1998). Only 10 (USP2, USP11, USP16, USP18, USP19, USP20, USP23, USP29, USP30, and USP35) out of 37 USPs found in *H. mediterranei* genome were predicted to contain an ATP-binding site due to the high sequence homology to *M. jannaschii* USP. The alignment shows the motif G2xG9xG(S/T) in almost all the USPs, except USP11 and USP18. In addition, ATP-binding motif residues indicate that ATP may regulate USPs. Considering this, *H. mediterranei* has 10 USPs that should be classified as proteins with a possible ATP-binding site, and 27 USPs without this site. Despite the above, further experimental procedures will be necessary to confirm whether these proteins bind ATP at the ATP binding site or not to improve this classification, as it is known that in other microorganisms such as *E. coli,* UspA does not bind ATP, whereas UspGF does (Zarembinski et al. 1998; Sousa and McKay 2001; Saveanu et al. 2002).

### Transcriptional expression of USP under different stress conditions

**Fig. 3:**
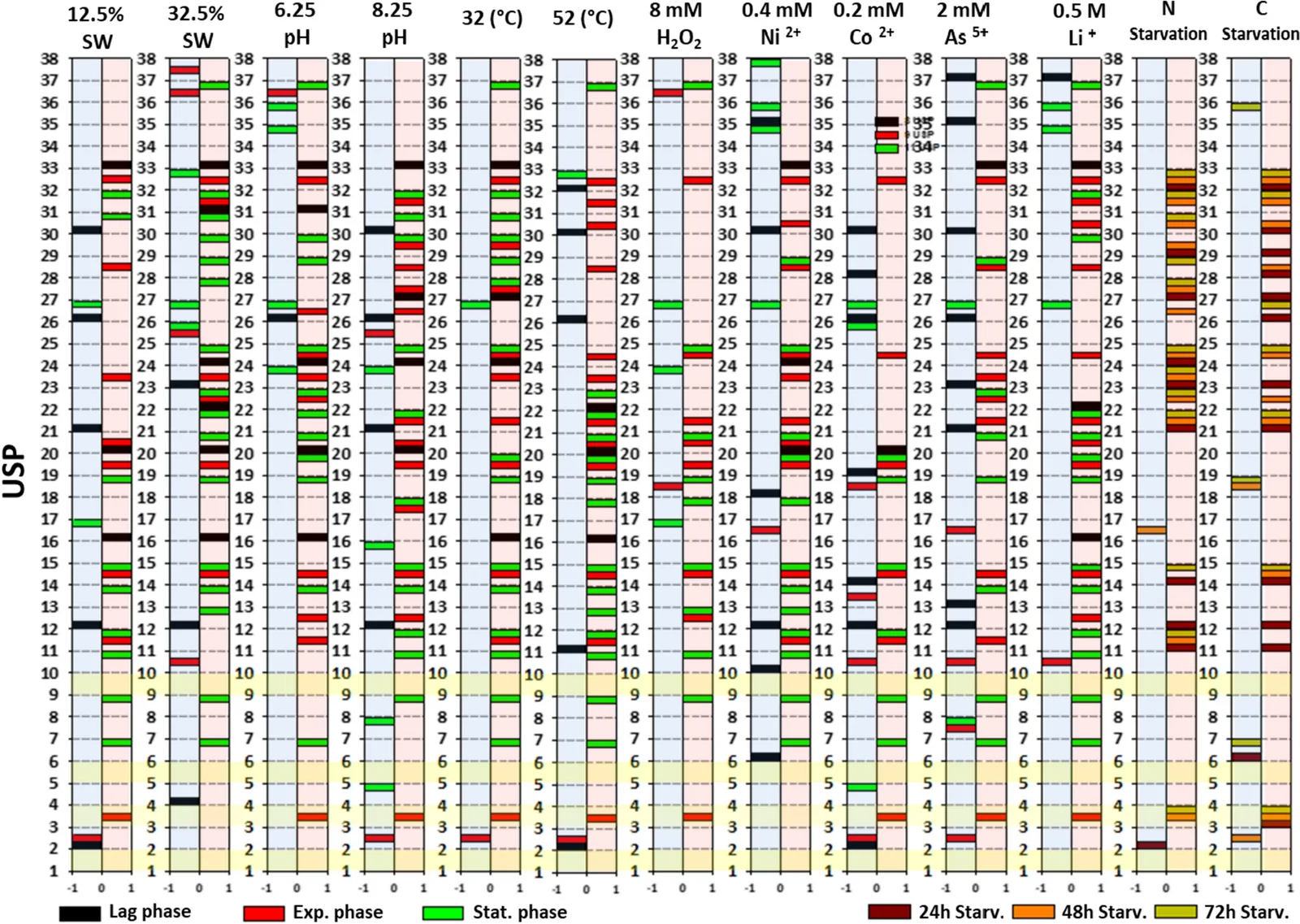
Expression patterns of the 37 analyzed USPs across various tested stressful conditions and different growth stages. On the abscissa axis, 1 and -1 indicate presence or absence of a USP, respectively, in comparison to the control condition. Proteins highlighted in yellow represent the steady stress proteins detected consistently across all tested conditions

Different studies revealed that the expression of USP depends on the stress type and growth phase. For example, *E. coli* UspA production was more related to the growth phase than the growth rate, as its transcriptional induction occurs whenever conditions lead to growth arrest (Freestone et al. 1997). Reverse-Transcriptase PCR (RT-PCR) was used to analyze the expression in different conditions of all 37 USPs identified in *H. mediterranei*. This analysis used RNA from cells grown under different stress conditions (temperature, salinity, pH, oxidative stress, metal stress, N starvation, and C starvation) in the three different growth phases to determine the transcriptional expression of all 37 identified USPs. The stress conditions have been chosen based on previous work (Matarredona et al. 2021b). The analysis of alterations in gene expression for each USP and stress condition was analysed through RT-PCR and agarose gel electrophoresis in three different growth phases. The summarized results presented in Table S4, S5, S6, and S7 are visually depicted in Fig. 3. As expected, the gene exhibited varying expression patterns under the tested stress conditions and through the time of culture.

**Fig. 4:**
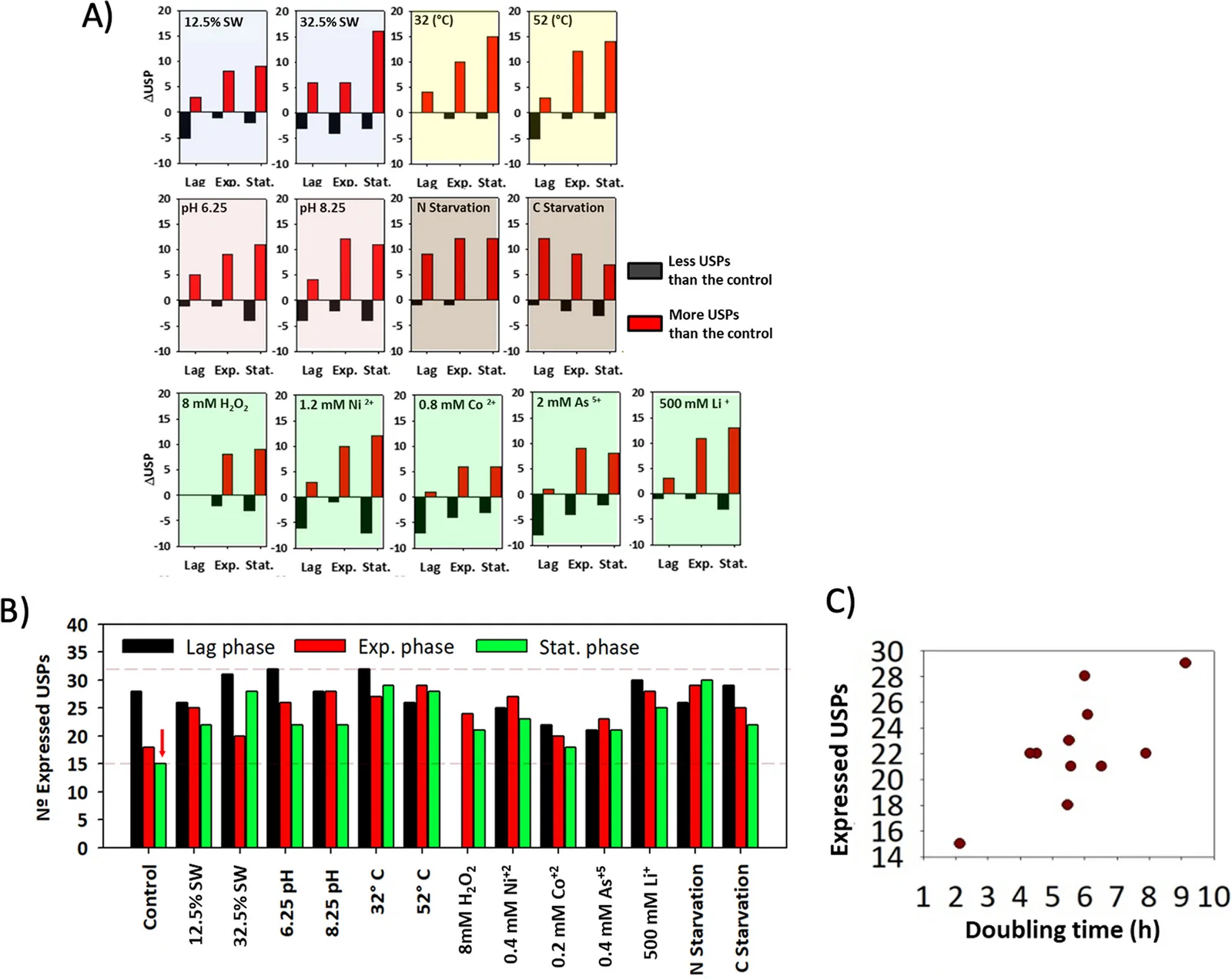
**A**) Temporal progression of the presence or absence of USPs concerning the control assays for each stressful condition. **B**) The absolute number of expressed USPs in each tested condition at various growth phases, alongside the doubling time of the culture. The red arrow indicates the lowest detected value of USPs. **C**) The relationship between the number of USPs expressed at the end of the growth and the corresponding duplication time figured

Preliminary analysis of these results reveals a significant overall increase in the presence of USPs under all tested stressful conditions. Clearly, this activation becomes notably pronounced during the consecutive stages of the growth: lag, exponential and stationary phases (Fig. 4 A). Under closer examination, we discovered that this substantial appearance of USPs in stress assays is a response to the down-regulation of USPs in the control test during the exponential and stationary phases (Fig. 4 B, red arrow). In the control condition, a considerable number of USPs (28 out of 37) were expressed in the lag phase, likely due to the stress of the culture adapting to a fresh medium. However, when cells recognized and adapted to a suitable (low-stress) environment, a large number of USPs were repressed to expedite growth, drastically reducing the number of expressed USPs (15 out of 37). In fact, a direct correlation was observed between the doubling times and the increasing number of USPs present in the metabolism (Fig. 4 C). This correlation started at 2.13 h (Matarredona et al. 2021b) with 15 USPs in control assay and concluded at 9.13 h with 32 UPSs under low temperature conditions.

**Fig. 5:**
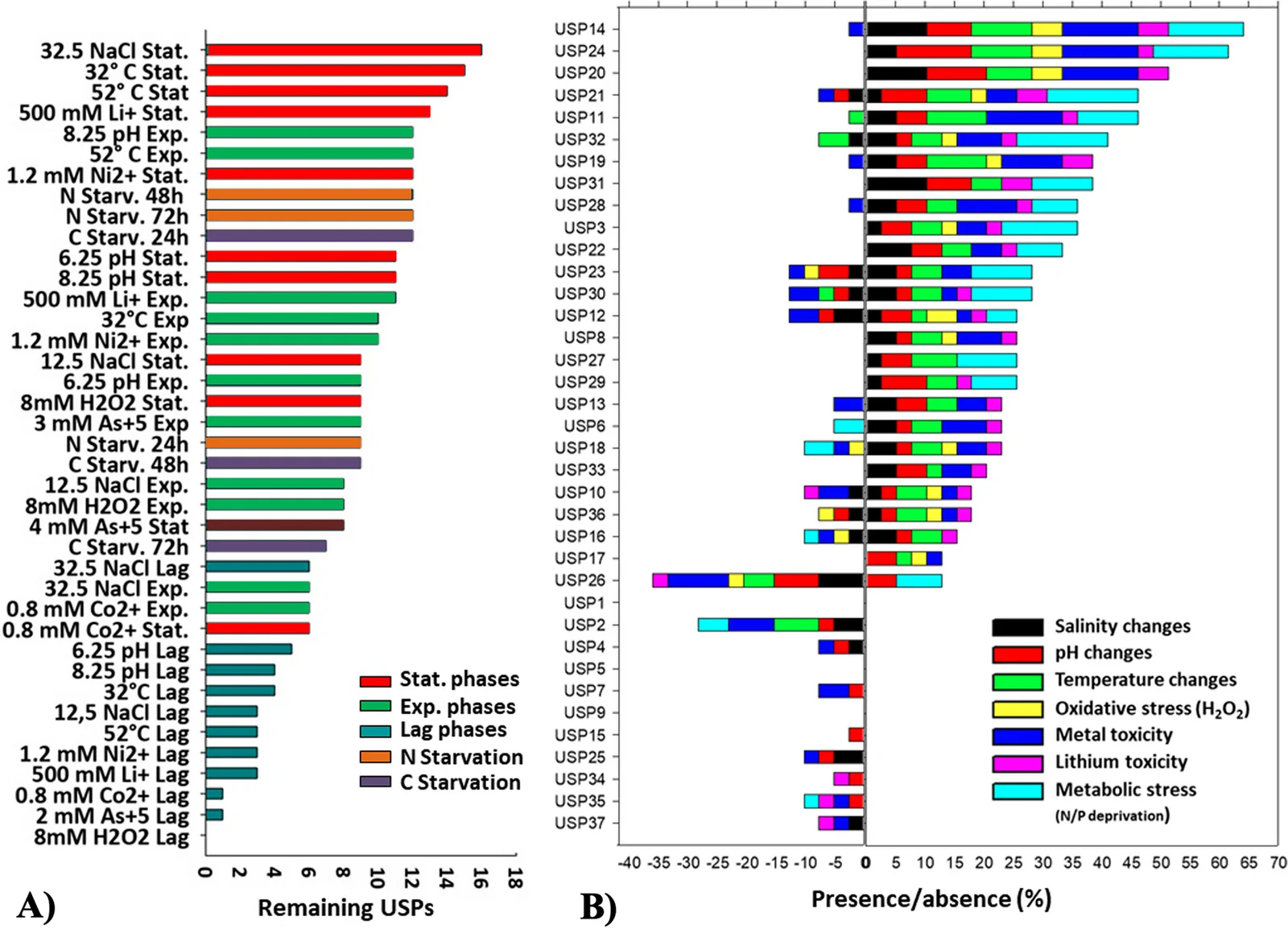
**A**) Bar ranking illustrating preserved USPs compared to the control for each specific type of stress. **B**) Bar ranking of the presence (positive axis) and absence (negative axis) of each single USP with respect to the control condition across the 39 tested situations. Stat: Stationary phase; Exp: Exponential phase; Lag: Initial exponential phase

Regarding individual sources of stress, the collected data revealed that extreme temperatures (32 and 52 °C), high salinity (32.5%) and high lithium concentrations (0.5 M) were the most stressful conditions. These conditions maintained the expression of more USPs (between 13 and 16) compared to the repression detected in the control condition (Fig. 5 A). Metabolic stress induced by nitrogen and carbon starvation also led to a strong presence of USPs (between 9 and 12). However, nitrogen starvation caused progressive stress, reaching its peak at 72 h after the depletion, while carbon starvation resulted in high UPS presence in the initial hours after the starvation, followed by a reduction in these stress signals, presumably due to metabolic slowdown.

In terms of individual stress proteins, USP14 is the protein more strongly present regarding all the tested condition, being detected in 25 out of 39 tested conditions (64%) compared to the control condition. In contrast, differences in the expression of USP26 are detected in 15 out of 39 tests (38.5%) compared to the control condition, becoming it the most frequently underexpressed protein (Fig. 5 B) with a significant difference in the frequency compared to the other USPs. Although most USPs exhibited a general response independent of the stress sources (USP14, 24, 21, etc.), it was possible to identify particular USPs associated with individual stress sources. For example, the expression of USP26 was detected only under nitrogen and carbon starvation or acidic pH conditions. Conversely, the down-regulation of different USPs (presents under control conditions but absent in applied stresses) appeared to be a more specific signal for the different stresses. Among them, the disappearance of USP7 seemed to be related to the presence of toxic metals, the loss of USP6 was detected only in metabolic stress assays, and the loss of USP26 could be associated with salinity stress. Lastly, it is worth noting the expression of three proteins (USP1, USP5 and USP9) in all tested conditions, regardless of the applied stress factor or growth phase (Fig. 5). These USPs seem to be constitutive across different metabolic stated displayed and could be termed as "Steady Universal Stress Proteins."

### RNA-Seq results

**Fig. 6:**
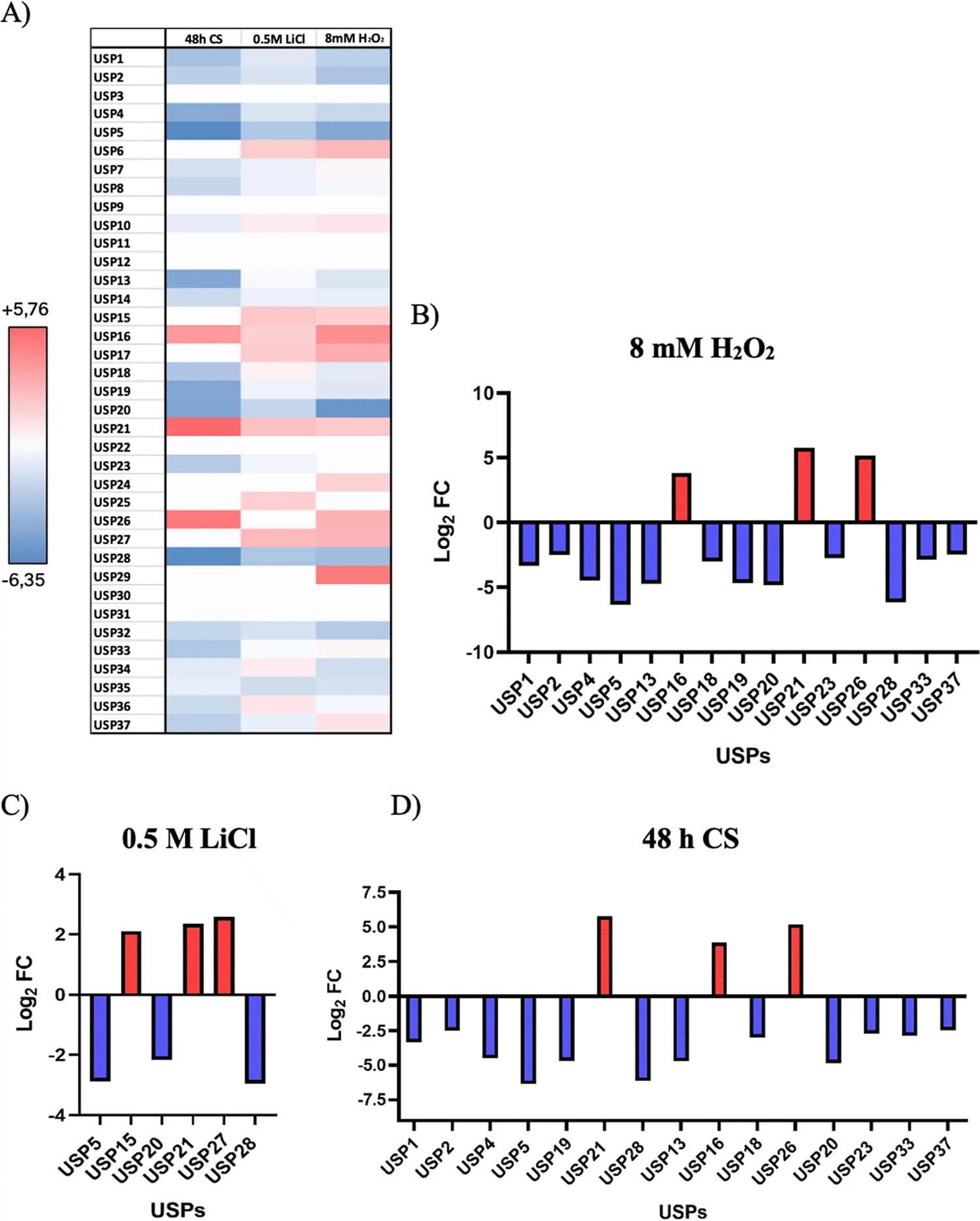
Analysis of all annotated UPSs in *H. mediterranei*. **A**) Heat map in the three-comparison contrast: 48 h CS-Control, 0.5 M LiCl-Control, and 8 mM H_2_O_2_-Control; **B**) USPs with significant expression differences in 8 mM H_2_O_2_; **C**) USPs with significant expression differences in 0.5 M LiCl; **D**) USPs with significant expression differences in 48 h CS

The transcriptomes of the three stress cultures (500 mM lithium, 8 mM H_2_O_2_, and 48 h carbon starvation) have been compared with the *H. mediterranei* cells grown under control conditions (Hm-DM; control) in mid-exponential phase for obtaining information on the effect of different stress conditions on *H. mediterranei* USP gene expression. Between 8.23 × 10^6^ and 1.25 × 10^7^ forward reads were obtained for each of the eight samples, and the total number was reduced to between 6.14 × 10^5^ and 4.43 × 10^6^ mRNA reads, after excluding reads mapping to rRNA genes (Table S8). These media were chosen because of the high number of USPs expressed in the RT-PCR, and also due to other studies performed in the research group (Matarredona et al. 2021b, 2021a). These two analyses, both RT-PCR and RNA-seq, are complementary although not easily comparably. The latter is more sensitive than RT-PCR nonetheless it does not offer evidence of absolute quantities about the contrasted transcripts. The results of the differential USPs expression analysis are shown in the following heat map (Fig. 6 A). Of all USPs, those that showed significant differences are shown in the Fig. 6 B-D. It should be noted that most of the USPs with differences were down-regulated. A total of 30 out of 37 USPs showed differences in expression at least one of the stress conditions tested. In the first analyzed condition, the 48 h CS-Control contrast showed 14 USPs with no significant differences, only three proteins up-regulated (USP16 shows a log_2_FC 3.85; USP21, a log_2_FC 5.76; and USP26, a log_2_FC 5.18) and the rest 20, down-regulated (USP5 with a log_2_FC -6.35; and USP28 with log_2_FC -6.14, proved to have the most relevant results). In the second condition, the 0.5 M LiCl-Control contrast showed 12 USPs without significance, 11 up-regulated (from log_2_FC 0.65 to log_2_FC 2.59), and the rest 14, down-regulated (from log_2_FC -0.67 to log_2_FC -2.95); but as shown in the heat map in this condition, the values were not so different, the numeric differences in the figure are not as intense as in the other two stress conditions. In the third condition, the 8 mM H_2_O_2_-Control contrast showed 11 USPs with no significant differences. Notably, USP5 (defined as steady USP in RT-PCR analysis) and USP20 were down-regulated, although not complete repression, as evidenced in Fig. 5 B, with log_2_FC -4.65 and -5.63, respectively. Conversely, USP16 and USP29 were up-regulated, showing log_2_FC 4.34 and 5.09, respectively.

Based on the RNA-Seq results, three stress proteins (USP5, USP21 and USP28) with marked differential expression compared to the control (Hm-DM) were selected. USP5 showed a log_2_FC -6.35 in 48 h CS, a log_2_FC -2.88 in 0.5 M lithium, and a log_2_FC -4.54 in 8 mM H_2_O_2_; this protein is down-regulated in these three conditions although it is considered as a steady USP protein from the RT-PCR analyses. In contrast, USP21 only showed differential expression in 48 h CS with a log_2_FC + 5.76. This fact agrees with RT-PCR results, where it was the fourth USP whose expression was detected under more tested conditions compared to the control (Fig. 5 b), highlighting its significant role in metabolic stress responses. This result indicates that USP21 is not as repressed in the control sample as suggested by RT-PCR, highlighting the higher sensibility of -RNA-seq over RT-PCR. USP28 showed a log_2_FC -6.14, -2.95, and -5.09 in 48 h CS, 0.5 M lithium, and 8 mM H_2_O_2_, respectively. This indicates that, as average, it was the most strongly repressed protein among the three contrasts analyzed by RNA-Seq.

### Purification and determination of molecular mass of USPs in *H. mediterranei *HM26

As described above, three USPs were selected based on RNA-Seq results. USP5 and USP28 were selected because they were always down-regulated in the contrast analyzed, while USP21 was always up-regulated. The three stress proteins were overexpressed using a native expression system (Allers et al. 2010). The *usp5, usp21,* and *usp28* genes were cloned independently into the pTA1992 plasmid that contains the histidine tag (6xHis tag) in the N-terminal site. The USP5, USP21, and USP28 were overexpressed in *H. mediterranei* HM26, and the purification procedure involved two chromatographic steps. The overexpression and subsequent purification were analyzed on a 14% polyacrylamide gel (Figure S1), yielding bands at 23, 16, and 18 kDa corresponding to the USP5, USP21 an USP28, respectively. The molecular mass of these three proteins was experimentally determined by gel filtration chromatography showed that the most feasible structure is a dimeric structure of approximately 46 (USP5), 32 (USP21), and 36 (USP28) kDa. Standard proteins used as markers to estimate this result and chromatograms obtained are shown in Figure S2.

### Phenotypic characterization of overexpression strains *H. mediterranei *HM26 USP5-HM26, USP21-HM26 and USP28-HM26

The phenotypic characterization of the three overexpression strains (USP5-HM26, USP21-HM26, and USP28-HM26) was performed by analyzing its growth compared with the control strain *H. mediterranei* HM26 in the same culture conditions as in the RT-PCR assay. In general, the growth curve of the three overexpression strains and the control strain HM26 was very similar in the presence of ammonium (Figure S3a), 8.25 pH (Figure S3b), 32 ºC (Figure S3c), and 52 ºC (Figure S3d). Furthermore, statistical analysis of the growth parameters (Table S9) revealed no significant differences between the growth of the parental strain HM26 and the three overexpression strains under these conditions. According to these results, both the effect of high and low temperature and basic pH do not affect strains growth. More extreme values should be tested.

**Fig. 7:**
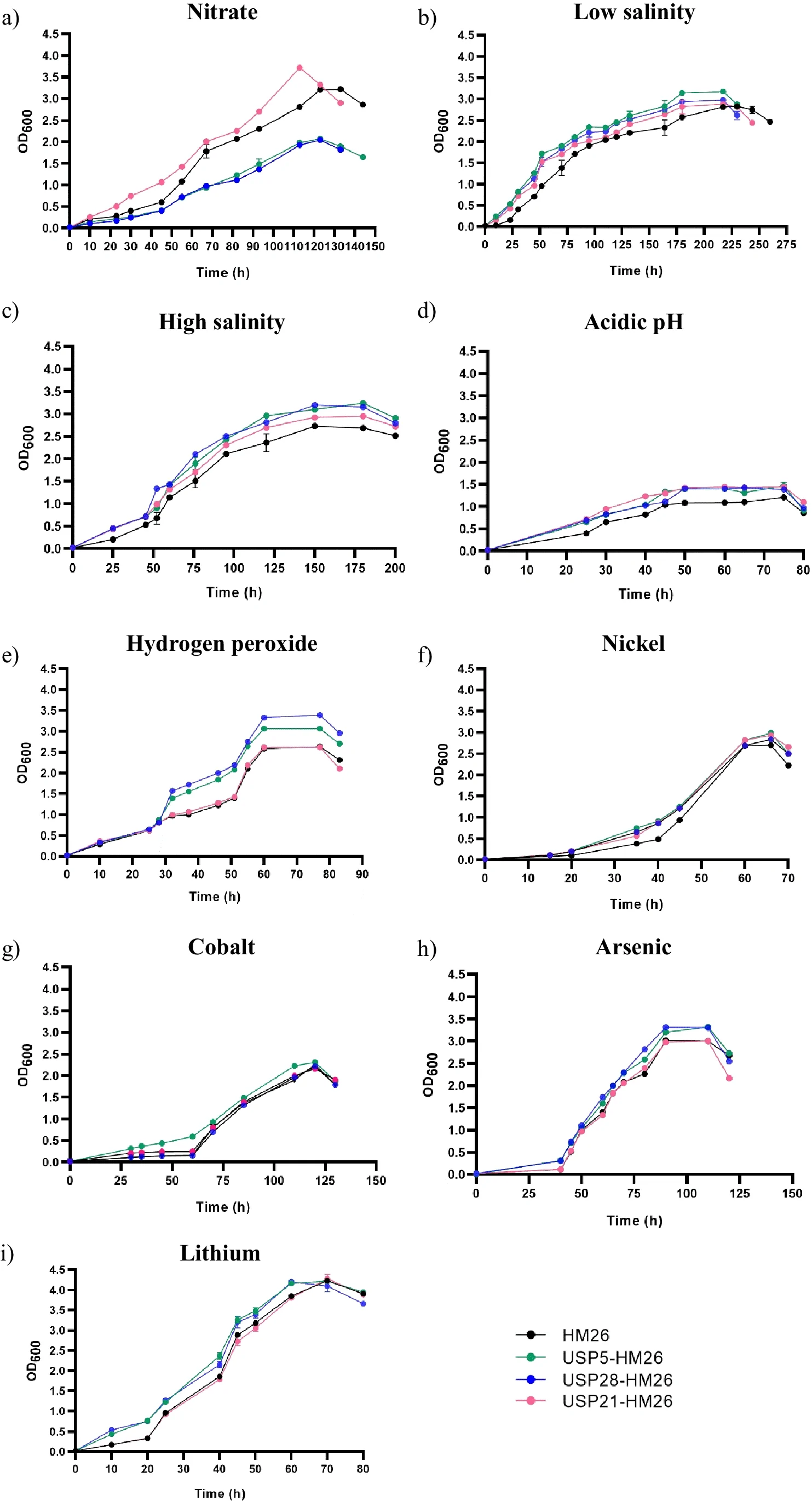
Growth of *H. mediterranei* HM26 and the three overexpression strains, USP5-HM26, USP21-HM26 and USP28-HM26 under different conditions. (**a**) 20 mM KNO_3_; (**b**) 12.5% SW; (**c**) 32.5% SW; (**d**) 6.25 pH; (**e**) 8 mM H_2_O_2_; (**f**) 0.4 mM Ni^2+^; (**g**) 0.2 mM Co^2+^; (**h**) 2 mM As^5+^; (**i**) 0.5 M Li^+^. In black: HM26; in green: USP5-HM25; in blue: USP28-HM26; and in pink: USP21-HM26. Data are based on three independent replicates. Plotted values are the mean of triplicate measurements, and error bars represent ± SD

Cell growth in nitrate, considered as N-limiting conditions, showed interesting results (Fig. 7 a). When USP5 is overexpressed, *H. mediterranei* grew better than the control (HM26) in nitrate; however, when USP21 and USP28 are overexpressed, the observed effect is the opposite. Up to now, there is not any work which associates the USPs with nitrogen metabolisms. However, analysis of *H. mediterranei* transcriptome (Esclapez et al. 2015) reveals that USP28-HM26 shows decreased expression in nitrogen starvation (48 h) in comparison to the control conditions. Consequently, this evidence suggests that USP28 could be involved in nitrogen metabolisms. More work is needed to elucidate the role of USPs in the nitrate assimilation metabolism.

Focusing on the response to changes in salinity concentration, the strains were exposed to hypo- (12.5% SW) and hypersaline (32.5%) stress taking into account that the control culture is grown at 20% SW. Comparison of the growth curves at 12.5% SW (Fig. 7 b) and 32.5% SW (Fig. 7 c) showed a similar trend, with the overexpression strains reaching higher OD_600_ values throughout all growth phases. Moreover, the doubling times of cultures were lower than the control, not only at low salt concentrations but also at high concentrations (Table S9). These results are consistent with previous findings obtained by RT-PCR, indicating that 32.5% was one of the most stressful conditions for *H. mediterranei*, leading to high expression of USPs.

The effect of pH changes on the growth of Universal Stress Proteins was also studied. At acidic pH such as 6.25 pH (Fig. 7 d), the growth of the overexpression strains showed higher optical density values, not considerably different from the control condition, but the analysis of their growth times was significantly lower than the control. However, as described above, pH 8.25 (Figure S3b) did not change the growth pattern of the USPs.

To study the effect of oxidative stress on the USP phenotypic characterization, the four *H. mediterranei* strains were exposed to 8 mM H_2_O_2_ in the mid-exponential phase (Fig. 7 e). A significant difference was detected in the growth of USP5-HM26 and USP28-HM26 strains; they grew much faster, showing changes in specific growth rates and doubling times, and reached higher values in the stationary phase. Strain USP21-HM26 followed the same trend as the parental strain. These data are both surprising and expected because, from the RNA-Seq data, we knew that USP21 showed no differences; however, USP5 was down-regulated with a log_2_ FC -4.5372, and USP28 was up-regulated with a log_2_ FC + 5.0914, and both grow much better than the control. Remarkably, even though one is up-regulated and the other is down-regulated, the effect on the *H. mediterranei* growth is similar, both enhance growth in the presence hydrogen peroxide. More work is needed to understand the mechanism in which these proteins are involved in oxidative stress.

To analyze the sensitivity of the overexpression strains to metal stress, four different metals (Ni^2+^, Co^2+^, As^5+^, and Li^+^) were selected (Fig. 7 f-i). USP21-HM26 strain only showed slightly changes in the presence of nickel, which reached higher stationary phase values; as with the other metals, its growth was the same as the parental one. On the contrary, the USP5-HM26 overexpression strain did show significant differences with the four metals analyzed. In fact, it showed better tolerance in all cases, reaching higher densities and better growth times. Finally, the growth of strain USP28-HM26 did not change in the presence of cobalt with respect to the control since nickel, arsenic, and lithium showed a different growth than the control. Furthermore, although USP5 and USP28 were down-regulated in the transcriptomic assay, their growth patterns were better than the parental strain.

## Discussion

This study marks the first comprehensive analysis of all the Universal Stress Proteins (USPs) in a microorganism under varied stress conditions and throughout its growth to the stationary phase. Notably, in the Archaea Domain, this research represents the pioneering exploration of USPs at molecular level. The lag phase, common to both control and stressful conditions, emerged as a potent source of stress. During this phase, cells must swiftly recognize and adapt to new environments, leading to the expression of a high number of USPs that deaccelerate the growth rate.

Following the lag phase, diverse conditions triggered distinct metabolic responses. In the control condition, where the environment was more favorable, nearly 50% of USPs transcripts were undetectable during exponential phase, leading to an accelerated duplication time of 2.1 h. Conversely, other conditions where a source of stress was present maintained the expression of a higher number of USPs compared to the control condition. However, despite this clear relationship between number of USPs and growth, it was difficult to associate the expression of a single USP with a particular type of stress. On the contrary, approximately one-third of all USPs remained expressed regardless of the stressor. All these results suggested that the USP system in *H. mediterranei* functions as a recruitment mechanism, where the number of stress proteins increases depending on the strength of the stressful stimulus. Different USPs exhibited specific sensibility to stress level. Indeed, USP14, USP24, and USP20 were the most consistently expressed in all the conditions, indicating their high sensibility to the stress. On the contrary, only two proteins (USP28 and USP2) were significantly repressed under stressful conditions, regardless of the stress source. Furthermore, several USPs in *H. mediterranei* (USP1, USP5, and USP9) exhibited universal responses to various stresses, designating them as steady universal stress proteins with a very high sensibility. Beyond the continuous presence of these USPs, RNA-Seq analysis revealed significant differences in the expression levels of USP5 between the tested conditions and the control. As overall idea, the basal expression of a minimal number of USPs could be attributed to the constant need for a high salt concentration, crucial for their survival of halophilic organisms like *H. mediterranei*. In fact, there are examples in other microorganisms where the expression of USPs is related to the salinity. In *S. acidocaldarius*, a thermoacidophilic archaeon, the expression of a universal stress protein (*Sa*UspA) was also studied under various environmental stress conditions. Stress conditions tested, such as starvation, high salinity, and UV stress, did not stimulate the expression of Δ*Sa*UspA. However, the UspA mutant showed impaired growth under high salinity stress conditions (Ye et al. 2020). In *M. portucalensis*, a UspA was believed to be expressed to repair DNA and proteins under salt stress conditions (Shih and Lai 2010).

Among all tested conditions, high temperature and salinity at the stationary phase emerged as the most stressful scenarios. In these situations, cells expressed 14–16 more USPs than the control under similar circumstances, and the observed doubling times were between 2–3 times higher. It should be note that the culture grown at 52ºC, expressed 14 more USPs than the control at stationary phase but its duplication time was only 3.38 h. This incongruity could be explained by the fact that, although this high temperature is a strong source of stress, it inherently produces an increment in the velocity of the enzymatic pathways, potentially masking the role of USPs.

In addition to the expression analysis of all USPs in *H. mediterranei*, further analysis was performed by RNA-Seq. It was expected that most USPs would show up-regulated expression compared with the control in response to the analyzed stress conditions. However, these results suggest that not only are USP proteins overexpressed in the face of harmful conditions, but they may also be down-regulated, and thus show a negative value of fold-change. These findings confirmed that, despite the recruitment system elucidated though RT-PCR trials, there is a fine tuning in the expression levels when a USP is present, although the absolute amounts of the transcripts remain unknown. Moreover, considering that all the tested conditions, including the control, initiate growth with high levels of expressed USPs during the lag phase, establishing correlations for these down-regulations is challenging. Briefly, a total of 30, of the 37 USPs, showed differences in expression in almost any of the stress conditions tested; among them, three: USP5, USP21, and USP28 were selected for in-depth characterization. These proteins showed the most remarkable expression differences concerning ammonium (control).

In relation to the characterization of USP5, USP21 and USP28, their quaternary structure corresponds to dimeric structure with 46, 32 and 36 kDa, respectively. This native conformation is an expected result since other examples of universal stress proteins have the same native conformation. USP protein MJ0577 from *M. jannaschii* (Zarembinski et al. 1998), HI0815 from *H. influenzae* Rv2623 (Sousa and McKay 2001), a USP protein from *Mycobacterium tuberculosis*(Drumm et al. 2009), and USP TTHA0350 from *Thermus thermophilus* HB8 have a dimer conformation (Iino et al. 2011). Other examples include USP AF0826 from *Archaeoglobus fulgidus* and a bacterial USP NE1028 from *Nitrosomonas europaea* (Tkaczuk et al. 2013), whose quaternary structure were determine by size-exclusion chromatography and dynamic light scattering; resulting as a dimeric structure.

Finally, the results of the phenotypic characterization of overexpression strains (USP5-HM26, USP21-HM26, and USP28-HM26), for all stress conditions tested, were in line with what was expected based on the previous classification of these proteins by RT-PCR. In the culture conditions where there was no expression of the proteins, no significant evidence of growth changes was found. Still, the strains showed differences or grew faster than expected in those conditions where USPs are expressed. Significant changes have been observed under nitrate, low and high salinities, 52 ºC, 6.25 pH; hydrogen peroxide, nickel, cobalt, arsenic and lithium. Of these, nitrogen starvation, hydrogen peroxide and salinity changes showed the greatest differences. The comparison of the results of both the phenotypic characterization and the growth rates of the overexpression strains of the three stress proteins suggests that the USP5 would be the best candidate to become *H. mediterranei* in a more tolerant strain to detrimental conditions. In fact, this protein was classified as steady stress protein hence its regulatory role should be strongly relevant. In the future, it would be interesting to analyze the expression of these USPs when *H. mediterranei* is subjected to several stresses simultaneously.

To sum up, although more work is needed to elucidate the molecular mechanisms of these universal stress proteins, this is a good starting point to improve applications of *H. mediterranei* in the field of biotechnology, such as the design of stress-resistant microorganisms.

## Supplementary Information

Below is the link to the electronic supplementary material.

Supplementary file1 (PDF 672 KB)

## Data Availability

The datasets generated during and/or analyzed during the current study are available from the corresponding author on reasonable request.
