# Supplementary material for: Deepening the knowledge of universal stress proteins in Haloferax mediterranei
Source: Appl Microbiol Biotechnol. 2024 Jan 16;108:124. doi: 10.1007/s00253-023-12899-1 (PMC10791855; doi:10.1007/s00253-023-12899-1)
Supplement: Supplementary file 1 [file 253_2023_12899_MOESM1_ESM.pdf]

Journal name: Applied Microbiology and Biotechnology

Manuscript title: Deepening the knowledge of universal stress proteins in *Haloferax mediterranei*

The name(s) of the author(s): Laura Matarredona[1], Basilio Zafrilla[1], Esther Rubio-Portillo[2], M-José Bonete[1], Julia Esclapez[1*]

The affiliation(s) and address(es): [1]Department of Biochemistry and Molecular Biology and Soil Science and Agricultural Chemistry, Faculty of Science, University of Alicante, Ap 99, 03080 Alicante, Spain.
[2] Department of Physiology, Genetics and Microbiology, Faculty of Science, University of Alicante, Ap 99, 03080 Alicante, Spain.

***The email address, telephone and fax number of corresponding authors:**
julia.esclapez@ua.es
Telephone number: +34 965903400 extension 1382
Fax number: +34 965909955

Table S1. Primer sequences for *H. mediterranei* genes encoding universal stress proteins used in RT-PCR.

	Primer	Sequence (5'→3')	bp	Tm(ºC)	%GC
**CONTROL (16S)**	**F**	CGTCCGCAAGGATGAAA	145	59.5	52.9
	**R**	CAGCGTCGTGGTAAGGT		61.1	58.8
**1**	**F**	ATGCCGGTCGTCGAAGACG	145	60.1	63.2
	**R**	CGCCCTGTGCTTCGAGTTC		58.9	63.2
**2**	**F**	TCCGAGGCAGTCCTCGAAC	145	59.2	63.2
	**R**	TCTCACCCTCGGTGGCAC		59.9	66.7
**3**	**F**	CTCGTTCCGATTGACGGCAC	146	58.6	60
	**R**	GCGACATCGTTGGAAGCGAAT		58.3	52.4
**4**	**F**	GGAAGTGCCCCCAGCGAAG	145	61.4	68.4
	**R**	CGTAGGCGCTCATGAGACCA		59.3	60
**5**	**F**	GCCAGTCCAGAAGCGGAGAA	145	59.6	60
	**R**	CTCGGTCACCGCGTTTGGAA		60.4	60
**6**	**F**	GACGATTCCATCCGAGCGCT	148	59.8	60
	**R**	CGCGTTCGTCGAGTACTTCC		58	60
**7**	**F**	GTTCTCGTGCCGGTTGACCA	145	60.2	60
	**R**	CGACGACGCCCTGCTCTATG		60.2	65
**8**	**F**	GGTTCGGCAAGCGTTCGC	145	60.4	66.7
	**R**	GTGCCTCGCGCATCTCATC		59	63.2
**9**	**F**	CGCAGCAATCGGAGAGGAAC	147	58.5	60
	**R**	GGGGTGGCTCTGATTGCCTC		60.7	65
**10**	**F**	TCATCCCCATCGACGGCACG	145	62.2	65
	**R**	CGAGCGTCGCTGAGGATAGC		60.2	65
**11**	**F**	GGCAGCGATGCAACCGAC	144	60.1	66.7
	**R**	CTTCCCACACGTCGGGGG		60.5	70.6
**12**	**F**	CGTTGTCCCGGTTGCGAAC	143	59.6	63.2
	**R**	GTTCAGCCTGTTCGACGGGG		60.8	65
**13**	**F**	ACTATCTTGCTCGCCGTTGG	146	57.5	55
	**R**	TTGTCGAGCGCGTCGTTGTA		59.3	55
**14**	**F**	AGTAGACGGGTCCGAGGCC	145	61.3	68.4
	**R**	TGCGACACCCACAACTGGC		61.1	63.2
**15**	**F**	CGGTGTCGATGACGACGTAG	146	57.5	60
	**R**	CCGAGTGAATCTGTGTCGCG		58.3	60
**16**	**F**	ATCCTCGTCGCGGTCGATG	148	59.7	63.2
	**R**	CGAGACTCACATCCACGTCG		57.5	60
**17**	**F**	TATCTCGTCGCCTACGACGG	152	58.4	60
	**R**	TTCTTCGGGACCCACCCCG		62.2	68.4
**18**	**F**	CACGCAATCGACATCGCCAG	149	59.2	60
	**R**	TCGATTGCCGTCTCGGCGTC		62.4	65
**19**	**F**	ATGCAGGCAATCGAGCACG	145	58.7	57.9
	**R**	CGATGGCGTCGTCTCGAAGC		60.8	65
**20**	**F**	GGAAGCGAGTGCGCAGACG	145	61.7	68.4
	**R**	CGTCTCGGACTCGTTCGAGA		58.4	60
**21**	**F**	CCTCCCAGTTGATGGGAGTCC	145	59.5	61.9
	**R**	GAGGACGGTACCCCCAACGA		61.2	65
**22**	**F**	ACCTTCATGGTCCCGTTCGA	142	58.6	55
	**R**	ATCCCTGCTGGCGGAGGTA		61	63.2
**23**	**F**	GAACGCGCCATCGACCACG	145	61.9	68.4
	**R**	CCTCACCCTCACGTTCGAGG		59.8	65
**24**	**F**	ATCCGCTCTCGAAGCACTCC	145	59.3	60
	**R**	TCGACCGCCCGCTTGAGTTC		62.5	65

**25**	**F**	CCACGTCGTCAATTCGCACC	144	59.1	60
	**R**	GGGTCGTTTCCGCGGATGTA		59.8	60
**26**	**F**	ATCGACGGGTCAGACGGG	145	59.5	66.7
	**R**	CGACGCCTTCCCACGATG		58.9	66.7
**27**	**F**	GGACGCCCGAATCACGCTG	140	61.6	68.4
	**R**	CCTCGGATTTTACCTCGGCG		58.1	60
**28**	**F**	TCGAATCCTCCGCGAAGCAG	148	59.8	60
	**R**	CTGGTCGATGCCGTAGACGAC		59.7	61.9
**29**	**F**	AGACGGTAGTGACACCGCAG	145	59.2	60
	**R**	ATGGCTGCGCATCTCTTCCC		60.4	60
**30**	**F**	CACGCTATTGACCTCGCAGG	143	58.3	60
	**R**	ACGTTGTTGACGGCCCGTTC		60.9	60
**31**	**F**	GTGCCCGATATCGAGCGGC	145	61.2	68.4
	**R**	CCGCTCCGTACCTCTCTTCG		59.5	65
**32**	**F**	GAATTCGCGGAAGAGCAGGG	145	58.6	60
	**R**	CCGCCACCTCTTTAACGCA		58.1	57.9
**33**	**F**	TCCTCCTTCCCGTCGACGAG	145	60.8	65
	**R**	ACGTCGTTACTCACGACCGT		58.4	55
**34**	**F**	ATTCCGGACCAGGTGATACGC	146	59.3	57.1
	**R**	GCCTCTACGAGGTCTTCGAGT		58.1	57.1
**35**	**F**	GGGAGCGAAACAGCGGAGT	145	60.3	63.2
	**R**	CTTGCCGTATGCGGTCGAC		58.9	63.2
**36**	**F**	GAGCGCGGTGTCGAGTACG	145	60.7	68.4
	**R**	CGTGGTCCTCAATCTGCTCG		58	60
**37**	**F**	GAAGCGGCAGTTGTCCGG	143	59.5	66.7
	**R**	CGCCGCTCGGAGTAGTTCC		60.5	68.4

Table S2. Primer sequences used in the amplification of the *H. mediterranei usp5, usp21* and *usp28* genes for the overexpression constructions.

	**Primer**	**Sequence (5’→3’)**	**bp**	**Tm(ºC)**	**%GC**
usp5	**F**	CCAGGAGGGTCAACATGTACAA	612	60.3	50
	**R**	GCGTTGGAATTCTCACCCGAC		61.8	57
usp21	**F**	GAGACATGTACGACAACATTCT	426	56.5	41
	**R**	CTTCTTCGAATTCTTACTCCGAA		56.5	39
usp28	**F**	CAGGACATGTAACGAGCACTC	498	59.8	52
	**R**	TCTTGGAATTCTTACGCGAGC		57.9	48

Table S3. General features of all annotated USPs in *H. mediterranei*.

USP	Accession code	Protein length (aa)	Domain position	Domain length (aa)	USP	Accession code	Protein length (aa)	Domain position	Domain length (aa)
**1**	WP_004059698.1	142	1-137	137	**20**	WP_004572467.1	145	1-141	141
**2**	WP_004059608.1	293	1-138<br>149-287	138<br>139	**21**	WP_004572399.1	141	1-138	138
**3**	WP_004059572.1	145	1-138	138	**22**	WP_004057007.1	163	2-140	139
**4**	WP_004059274.1	148	1-139	139	**23**	WP_004056910.1	162	1-139	139
**5**	WP_004059268.1	203	1-132	132	**24**	WP_004060178.1	135	2-132	131
**6**	WP_004059226.1	122	2-122	121	**25**	WP_004060004.1	134	65-129	65
**7**	WP_004059223.1	151	11-150	140	**26**	WP_004059939.1	184	4-143	140
**8**	WP_179955348.1	153	1-137	137	**27**	WP_004059784.1	141	1-141	141
**9**	WP_004059171.1	148	2-143	142	**28**	WP_004059756.1	165	2-145	144
**10**	WP_004058841.1	129	3-129	127	**29**	WP_004060603.1	290	1-138<br>147-283	138<br>137
**11**	WP_004058785.1	146	1-141	141	**30**	WP_004060612.1	151	1-139	139
**12**	WP_004058692.1	138	7-132	126	**31**	WP_004060615.1	142	5-142	138
**13**	WP_004058110.1	152	4-148	145	**32**	WP_004060695.1	139	3-135	133
**14**	WP_004058053.1	149	2-149	148	**33**	WP_004060722.1	289	1-136<br>149-283	136<br>135
**15**	WP_004057353.1	134	1-127	127	**34**	WP_004060964.1	281	133-235	102
**16**	WP_004572739.1	147	3-147	145	**35**	WP_004056272.1	154	1-147	147
**17**	WP_004572735.1	158	3-116	114	**36**	WP_004056137.1	140	1-138	138
**18**	WP_004572725.1	143	1-142	142	**37**	WP_004056131.1	292	1-141<br>155-289	141<br>135
**19**	WP_004572580.1	142	1-139	139					

Table S4. Summarize of USP expression in lag phase analyzed by RT-PCR*.

USP	Control	12.5% SW	32.5% SW	6.25 pH	8.25 pH	32°C	52°C	8 mM H_2O_2	0.4 mM Ni^{2+}	0.2 mM Co^{2+}	2 mM As^{5+}	500 mM Li^{+}
1	1	1	1	1	1	1	1	0	1	1	1	1
2	1	0	1	1	1	1	0	0	1	0	1	1
3	1	1	1	1	1	1	1	0	1	1	1	1
4	1	1	0	1	1	1	1	0	1	1	1	1
5	1	1	1	1	1	1	1	0	1	1	1	1
6	1	1	1	1	1	1	1	0	0	1	1	1
7	1	1	1	1	1	1	1	0	1	1	1	1
8	1	1	1	1	1	1	1	0	1	1	1	1
9	1	1	1	1	1	1	1	0	1	1	1	1
10	1	1	1	1	1	1	1	0	0	1	1	1
11	1	1	1	1	1	1	0	0	1	1	1	1
12	1	0	0	1	0	1	1	0	0	0	0	1
13	1	1	1	1	1	1	1	0	1	1	0	1
14	1	1	1	1	1	1	1	0	1	0	1	1
15	1	1	1	1	1	1	1	0	1	1	1	1
16	0	1	1	1	0	1	1	0	0	0	0	1
17	0	0	0	0	0	0	0	0	0	0	0	0
18	1	1	1	1	1	1	1	0	0	1	1	1
19	1	1	1	1	1	1	1	0	1	0	1	1
20	0	1	1	1	1	0	1	0	1	1	0	0
21	1	0	1	1	0	1	1	0	1	1	0	1
22	0	0	1	0	0	0	1	0	0	0	0	1
23	1	1	0	1	1	1	1	0	1	1	0	1
24	0	0	1	1	1	1	0	0	1	0	0	0
25	1	1	1	1	1	1	1	0	1	1	1	1
26	1	0	1	0	0	1	0	0	1	0	0	1
27	0	0	0	0	1	1	0	0	0	0	0	0
28	1	1	1	1	1	1	1	0	1	0	1	1
29	0	0	0	0	0	0	0	0	0	0	0	0
30	1	0	1	1	0	1	0	0	0	0	0	1
31	0	0	1	1	0	0	0	0	0	0	0	0
32	1	1	1	1	1	1	0	0	1	1	1	1
33	0	1	1	1	1	1	0	0	1	0	1	1
34	1	1	1	1	1	1	1	0	1	1	1	1
35	1	1	1	1	1	1	1	0	0	1	0	1
36	1	1	1	1	1	1	1	0	1	1	1	1
37	1	1	1	1	1	1	1	0	1	1	0	0
**Recruited**	28	26	31	32	28	32	26	0	25	22	21	30

*1 indicates the presence of expression, and 0 indicates the absence of expression.

Table S5. Summarize of USP expression in mid-exponential phase analyzed by RT-PCR *.

USP	Control	12.5% SW	32.5% SW	6.25 pH	8.25 pH	32°C	52°C	8 mM H_2O_2	0.4 mM Ni^{2+}	0.2 mM Co^{2+}	2 mM As^{5+}	500 mM Li^{+}
1	1	1	1	1	1	1	1	1	1	1	1	1
2	1	0	1	1	0	0	0	1	1	0	0	1
3	0	1	0	1	1	1	1	1	0	1	1	1
4	1	1	1	1	1	1	1	1	1	1	1	1
5	1	1	1	1	1	1	1	1	1	1	1	1
6	1	1	1	1	1	1	1	1	1	1	1	1
7	1	1	1	1	1	1	1	1	1	1	0	1
8	1	1	1	1	1	1	1	1	1	1	1	1
9	1	1	1	1	1	1	1	1	1	1	1	1
10	1	1	0	1	1	1	1	1	1	0	0	0
11	0	1	0	1	0	1	1	0	1	1	1	0
12	0	0	0	1	1	0	0	1	0	0	0	1
13	1	1	1	1	1	1	1	1	1	0	1	1
14	0	1	1	1	1	1	1	1	1	1	1	1
15	1	1	1	1	1	1	1	1	1	1	1	1
16	1	1	1	1	1	1	1	1	0	1	0	1
17	0	0	0	0	1	0	0	0	0	0	0	0
18	1	1	1	1	1	1	1	0	1	0	1	1
19	0	1	1	0	1	1	1	1	1	1	0	1
20	0	1	0	0	1	0	1	1	1	0	0	1
21	0	0	0	0	1	1	1	1	1	0	1	1
22	0	0	1	1	0	0	0	0	0	0	1	0
23	0	1	1	1	0	1	1	0	1	0	1	0
24	0	0	0	1	0	1	1	1	1	1	1	1
25	1	1	0	1	0	1	1	1	1	1	1	1
26	0	0	0	1	1	0	0	0	0	0	0	0
27	0	0	0	0	1	1	0	0	0	0	0	0
28	0	1	0	0	1	0	1	0	1	0	1	1
29	0	0	0	0	1	1	0	0	0	0	0	0
30	0	0	0	0	0	0	1	0	1	0	0	1
31	0	0	1	0	1	0	1	0	0	0	0	1
32	0	1	1	1	0	1	1	1	1	1	1	1
33	0	0	0	0	0	0	0	0	0	0	0	0
34	1	1	1	1	1	1	1	1	1	1	1	1
35	1	1	1	1	1	1	1	1	1	1	1	1
36	1	1	0	0	1	1	1	0	1	1	1	1
37	1	1	0	1	1	1	1	1	1	1	1	1
**Recruited**	18	25	20	26	28	27	29	24	27	20	23	28

*1 indicates the presence of expression, and 0 indicates the absence of expression.

Table S6. Summarize of USP expression in stationary phase analyzed by RT-PCR*.

USP	Control	12.5% SW	32.5% SW	6.25 pH	8.25 pH	32°C	52°C	8 mM H_2O_2	0.4 mM Ni^{2+}	0.2 mM Co^{2+}	2 mM As^{5+}	500 mM Li^{+}
1	1	1	1	1	1	1	1	1	1	1	1	1
2	0	0	0	0	0	0	0	0	0	0	0	0
3	1	1	1	1	1	1	1	1	1	1	1	1
4	1	1	1	1	0	1	1	1	1	0	1	1
5	1	1	1	1	1	1	1	1	1	1	1	1
6	0	1	1	1	0	1	1	0	1	1	1	1
7	1	1	1	1	0	1	1	1	1	1	0	1
8	0	1	1	0	1	1	1	1	1	1	1	1
9	1	1	1	1	1	1	1	1	1	1	1	1
10	0	1	0	0	1	1	1	1	1	0	0	1
11	0	1	0	0	1	1	1	0	1	1	0	1
12	0	0	1	0	0	0	1	1	1	0	0	0
13	0	1	1	1	1	1	1	0	1	0	1	1
14	0	1	1	0	1	1	1	1	1	1	0	1
15	1	1	1	1	0	1	1	1	1	1	1	1
16	1	0	1	1	1	1	1	0	1	1	1	1
17	0	0	0	0	1	0	1	1	1	0	0	0
18	0	1	1	1	0	1	1	1	0	1	1	1
19	0	0	0	1	0	1	1	0	1	1	0	1
20	0	0	1	1	0	0	1	1	1	0	1	1
21	0	0	1	1	1	0	1	0	0	0	0	1
22	0	0	1	1	0	0	1	0	0	0	1	0
23	1	1	1	0	0	1	1	0	1	1	1	1
24	0	0	1	1	1	1	0	1	1	0	0	0
25	1	1	0	1	1	1	1	1	1	0	1	1
26	1	0	0	0	1	0	1	0	0	0	0	0
27	0	0	1	0	0	1	0	0	0	0	0	0
28	0	0	1	1	0	1	0	0	1	0	1	0
29	0	0	1	1	1	1	0	0	0	0	0	1
30	0	1	1	0	1	1	0	0	0	0	0	0
31	0	1	1	0	1	1	0	0	0	0	0	1
32	1	1	0	1	1	1	0	1	1	1	1	1
33	0	0	0	0	0	0	0	0	0	0	0	0
34	1	1	1	0	1	1	1	1	0	1	1	0
35	1	1	1	0	1	1	1	1	0	1	1	0
36	0	0	1	1	0	1	1	1	0	0	1	1
37	1	1	1	1	1	1	1	1	0	1	1	1
Recruited	15	22	28	22	22	29	28	21	23	18	21	25

*1 indicates the presence of expression, and 0 indicates the absence of expression.

Table S7. Summarize of USP expression in Carbon and Nitrogen Starvation analyzed by RT-PCR*.

USP	NS 24h	NS 48h	NS 72 h	CS 24 h	CS 48h	CS 72 h
1	1	1	1	1	1	1
2	0	0	0	0	0	0
3	1	1	1	1	1	1
4	1	1	1	1	0	1
5	1	1	1	1	1	1
6	0	1	1	1	0	1
7	1	1	1	1	0	1
8	0	1	1	0	1	1
9	1	1	1	1	1	1
10	0	1	0	0	1	1
11	0	1	0	0	1	1
12	0	0	1	0	0	0
13	0	1	1	1	1	1
14	0	1	1	0	1	1
15	1	1	1	1	0	1
16	1	0	1	1	1	1
17	0	0	0	0	1	0
18	0	1	1	1	0	1
19	0	0	0	1	0	1
20	0	0	1	1	0	0
21	0	0	1	1	1	0
22	0	0	1	1	0	0
23	1	1	1	0	0	1
24	0	0	1	1	1	1
25	1	1	0	1	1	1
26	1	0	0	0	1	0
27	0	0	1	0	0	1
28	0	0	1	1	0	1
29	0	0	1	1	1	1
30	0	1	1	0	1	1
31	0	1	1	0	1	1
32	1	1	0	1	1	1
33	0	0	0	0	0	0
34	1	1	1	0	1	1
35	1	1	1	0	1	1
36	0	0	1	1	0	1
37	1	1	1	1	1	1
**Recruited**	15	22	28	22	22	29

*1 indicates the presence of expression, and 0 indicates the absence of expression.

Table S8. Transcriptomes genomic features.

	Number of seqs	seqs 16S rRNA & 23S rRNA	% rRNA	Mb mRNA
H_20_2 8mM_1	1.13E+07	8.38E+06	74.28	2.90E+06
H_20_2 8mM_2	1.03E+07	7.59E+06	73.52	2.73E+06
H_20_2 8mM_3	1.25E+07	8.46E+06	67.88	4.00E+06
CS 48h_1	9.13E+06	8.29E+06	90.76	8.44E+05
CS 48h_2	9.44E+06	8.73E+06	92.53	7.05E+05
CS 48h_3	8.23E+06	7.61E+06	92.54	6.14E+05
0.5 M LiCl_1	1.14E+07	7.33E+06	64.00	4.12E+06
0.5 M LiCl_2	1.07E+07	7.39E+06	68.96	3.33E+06
0.5 M LiCl_3	1.22E+07	7.80E+06	63.76	4.43E+06
20 mM NH_4Cl_1	1.13E+07	8.35E+06	74.04	2.93E+06
20 mM NH_4Cl _2	1.25E+07	8.55E+06	68.36	3.96E+06
20 mM NH_4Cl _3	9.88E+06	7.53E+06	76.25	2.35E+06

Table S9. Doubling time for *H. mediterranei* HM26, USP5-HM26, USP21-HM26 and USP28-HM26.

Condition	Strain	d.t. (h)*	p-value	Significance
20 mM NH_4Cl	HM26	2.13 ± 0.08		
	USP5-HM26	2.15± 0.08	>0.999	Ns
	USP21-HM26	2,10± 0.05	>0.999	Ns
	USP28-HM28	2.18± 0.07	>0.999	Ns
20 mM KNO_3	HM26	5.18 ± 0.04		
	USP5-HM26	6.84 ± 0.04	0.042	*
	USP21-HM26	4.57 ± 0.02	>0.999	Ns
	USP28-HM28	6.72 ± 0.06	0.039	*
12.5 % SW	HM26	7.9 ± 0.2		
	USP5-HM26	2.9 ± 0.1	<0.001	***
	USP21-HM26	3.7 ± 0.1	0.006	**
	USP28-HM28	2.9 ± 0.1	<0.001	***
32.5 % SW	HM26	6.01± 0.09		
	USP5-HM26	4.22±0.08	0.003	**
	USP21-HM26	5.68±0.09	>0.999	Ns
	USP28-HM28	3.38±0.09	0.004	**
6.25 pH	HM26	4.52± 0.02		
	USP5-HM26	2.30± 0.03	0.004	**
	USP21-HM26	2.28± 0.03	0.004	**
	USP28-HM28	2.26± 0.08	0.004	**
8.25 pH	HM26	4.30±0.09		
	USP5-HM26	4.32±0.09	>0.999	Ns
	USP21-HM26	4.28±0.08	>0.999	Ns
	USP28-HM28	4.25±0.09	>0.999	Ns
32 ºC	HM26	9.13±0.02		
	USP5-HM26	9.30±0.02	>0.999	Ns
	USP21-HM26	9.24±0.05	>0.999	Ns
	USP28-HM28	8.67±0.04	>0.999	Ns
52 ºC	HM26	3.38±0.09		
	USP5-HM26	2.34±0.09	>0.999	Ns
	USP21-HM26	2.58±0.09	>0.999	Ns
	USP28-HM28	3.38±0.09	>0.999	Ns
8 mM H_2O_2	HM26	5.58±0.01		
	USP5-HM26	2.25±0.09	0.004	**
	USP21-HM26	5.61±0.02	>0.999	Ns
	USP28-HM28	2.12±0.04	0.005	**
0.4 mM Ni^{2+}	HM26	5.52±0.09		
	USP5-HM26	4.25±0.09	0.002	*
	USP21-HM26	4.56±0.08	0.002	*
	USP28-HM28	4.40±0.09	0.002	*

0.2 mM Co^{2+}	HM26	5.47±0.04		
	USP5-HM26	4.87±0.01	0.002	*
	USP21-HM26	5.33±0.03	>0.999	Ns
	USP28-HM28	5.50±0.03	>0.999	Ns
2 mM As^{5+}	HM26	6.53±0.02		
	USP5-HM26	4.44±0.03	0.003	*
	USP21-HM26	6.32±0.03	>0.999	Ns
	USP28-HM28	4.56±0.02	0.003	*
0.5 M Li^{+}	HM26	6.10±0.01		
	USP5-HM26	2.25±0.01	0.006	**
	USP21-HM26	6.05±0.03	>0.999	Ns
	USP28-HM28	2.04±0.02	0.006	**

Ns: $p > 0.05$; * $p \leq 0.05$; ** $p \leq 0.01$; *** $p \leq 0.001$.

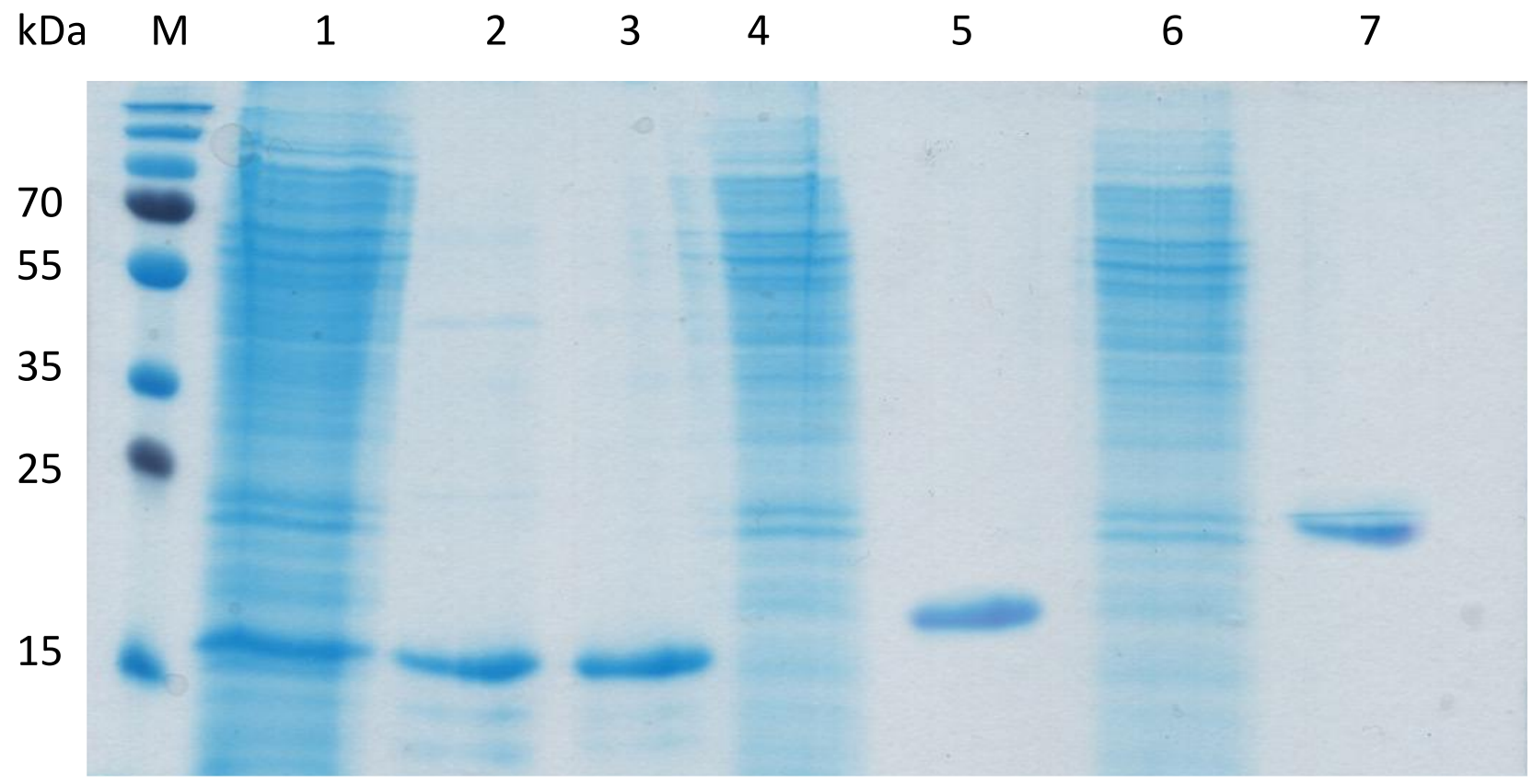

Figure S1. Homologous overexpression and purification of USP5, USP21, and USP28 in *H. mediterranei* HM26. 14 % SDS-PAGE with Coomassie Brilliant Blue staining. M: PageRuler Plus Prestained Protein Ladder (Thermo Fisher Scientific, Waltham, Massachusetts, USA); Line 1: Overexpression of USP5; Line 2: His-tagged fraction of USP5; Line 3: HiPrep 16/60 Sephacryl S-200 HR fraction of USP5; Line 4: Overexpression of USP21; Line 5: HiPrep 16/60 Sephacryl S-200 HR fraction of USP21; Line 6: Overexpression of USP28; Line 7: HiPrep 16/60 Sephacryl S-200 HR fraction of USP28.

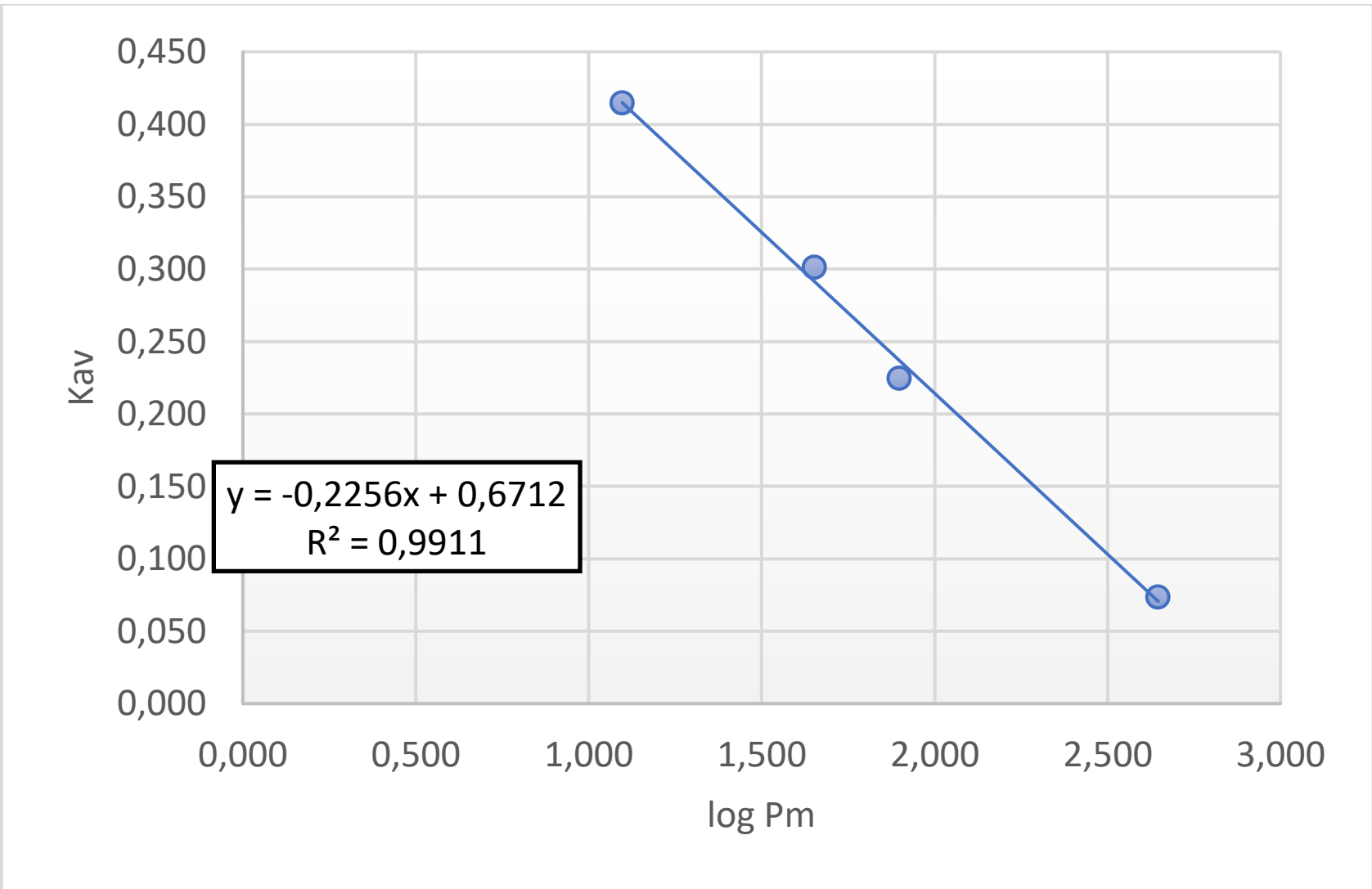

Figure S2. Size exclusion chromatography calibration curve for HiPrep 16/60 Sephacryl S-200 HR column.

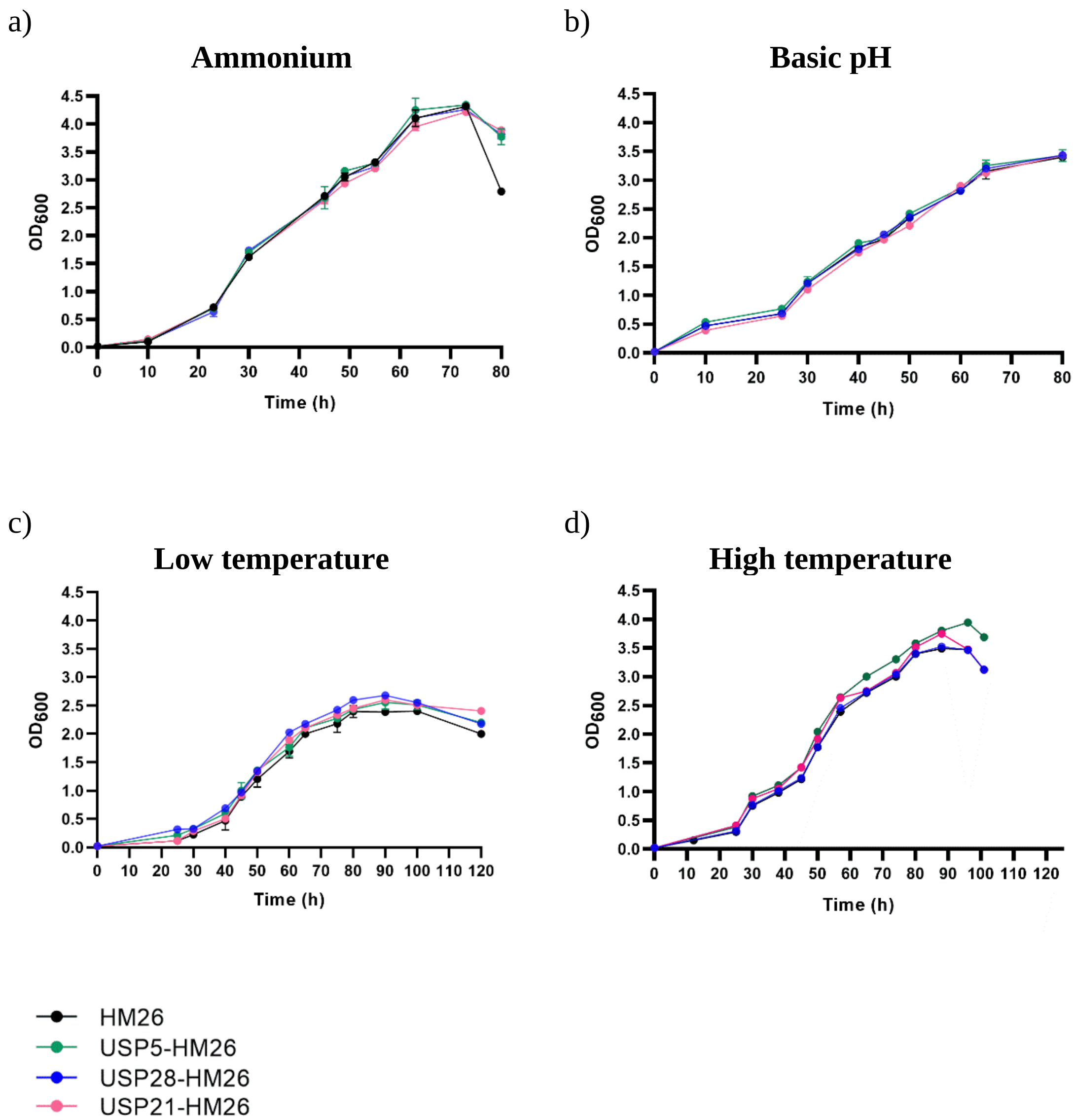

Figure S3. Growth of *H. mediterranei* HM26 and the three overexpression strains, USP5-HM26, USP21-HM26 and USP28-HM26 under different conditions. (a) 20 mM NH_4Cl; (b) 8.25 pH (c) 32 ºC; (d) 52 ºC. In black: HM26; in green: USP5-HM25; in blue: USP28-HM26; and in pink: USP21-HM26. Data are based on three independent replicates. Plotted values are the mean of triplicate measurements, and error bars represent ± SD.
